# Plasticity in Prefrontal Cortex Induced by Coordinated Synaptic Transmission Arising from Reuniens/Rhomboid Nuclei and Hippocampus

**DOI:** 10.1093/texcom/tgab029

**Published:** 2021-04-14

**Authors:** Paul J Banks, E Clea Warburton, Zafar I Bashir

**Affiliations:** School of Physiology, Pharmacology & Neuroscience, University of Bristol, Biomedical Sciences Building, University Walk, Bristol BS8 1TD, UK; School of Physiology, Pharmacology & Neuroscience, University of Bristol, Biomedical Sciences Building, University Walk, Bristol BS8 1TD, UK; School of Physiology, Pharmacology & Neuroscience, University of Bristol, Biomedical Sciences Building, University Walk, Bristol BS8 1TD, UK

**Keywords:** associative plasticity, neuromodulation, prefrontal cortex, short-term plasticity, thalamocortical

## Abstract

The nucleus reuniens and rhomboid nuclei of the thalamus (ReRh) are reciprocally connected to a range of higher order cortices including hippocampus (HPC) and medial prefrontal cortex (mPFC). The physiological function of ReRh is well predicted by requirement for interactions between mPFC and HPC, including associative recognition memory, spatial navigation, and working memory. Although anatomical and electrophysiological evidence suggests ReRh makes excitatory synapses in mPFC there is little data on the physiological properties of these projections, or whether ReRh and HPC target overlapping cell populations and, if so, how they interact. We demonstrate in ex vivo mPFC slices that ReRh and HPC afferent inputs converge onto more than two-thirds of layer 5 pyramidal neurons, show that ReRh, but not HPC, undergoes marked short-term plasticity during theta frequency transmission, and that HPC, but not ReRh, afferents are subject to neuromodulation by acetylcholine acting via muscarinic receptor M2. Finally, we demonstrate that pairing HPC followed by ReRh (but not pairing ReRh followed by HPC) at theta frequency induces associative, NMDA receptor dependent synaptic plasticity in both inputs to mPFC. These data provide vital physiological phenotypes of the synapses of this circuit and provide a novel mechanism for HPC–ReRh–mPFC encoding.

## Introduction

The medial prefrontal cortex (mPFC) is vital for performance of many higher order cognitive functions including decision-making, attention, and mnemonic processing. Evidence has emerged that communication between mPFC and the hippocampus (HPC), which results in synchronous oscillatory activity and cell firing (Jones and Wilson 2005; Siapas et al. 2005), is required for performance of some of these behaviors including during the encoding phase of spatial working memory (Spellman et al. 2015) and associative recognition memory (Barker et al. 2017).

More recently, regions of the ventral midline thalamus centered upon the adjacent reuniens and rhomboid nuclei (ReRh) have been shown to be involved in many cognitive processes which require HPC–mPFC interactions (reviewed by Dolleman-van der Weel et al. 2019), including encoding of associative recognition memory (Barker and Warburton 2018) and working memory (Hallock et al. 2016). ReRh has dense reciprocal connections to both HPC and mPFC and has been proposed to be a primary route of feedback from the mPFC to HPC (Cassel et al. 2013). ReRh relays trajectory information from mPFC to CA1 during spatial navigation (Ito et al. 2015). ReRh is also reciprocally connected to associative cortices including entorhinal and perirhinal cortex, which are themselves directly and indirectly connected to HPC and mPFC. Deactivation of ReRh reduces oscillatory coherence and phase-locking between HPC and mPFC during spatial working memory (Hallock et al. 2016), suggesting that ReRh is situated ideally as a long-range coordinator of higher order cortical structures.

Moreover, manipulations of ReRh produce complex delay-dependent effects on HPC–mPFC dependent memory, suggesting that ReRh does not act as a simple relay but is important for encoding memory by coordination of long-range connections (Barker and Warburton 2018). Indeed, the behavioral effects of blocking protein synthesis within ReRh suggest that plasticity processes may even encode and store memory within ReRh itself (Barker and Warburton 2018).

Acetylcholine signaling in mPFC is essential for associative recognition memory (Barker and Warburton 2009) and spatial working memory (Ragozzino and Kesner 1998), and additionally phasic ACh release in mPFC is associated with high cognitive load, cue-detection, and promotion of HPC-mPFC coherence (Howe et al. 2017; Teles-Grilo Ruivo et al. 2017). Given the importance of ReRh function in these behaviors and in regulation of oscillatory activity (Hallock et al. 2016; Barker and Warburton 2018) it is likely that ReRh inputs to mPFC are active during phasic ACh release in mPFC, however it is unknown whether ReRh afferents are sensitive to modulation by ACh. In addition, dopaminergic signaling in the mPFC plays a key role in executive function in mPFC (Ott and Nieder 2019) and, among other effects, dopamine is known to modulate synaptic transmission in mPFC, with varied effects upon local and distal excitatory synapses (Law-Tho et al. 1994; Gao et al. 2001; Seamans et al. 2001; Urban et al. 2002; Gonzalez-Islas and Hablitz 2003). However, it is not known how dopamine regulates ReRh evoked synaptic transmission in mPFC.

Although evidence for the functional importance of ReRh in cognitive processes is strong, very little is known about the nature of ReRh afferents to mPFC, or how they may interact with those of the HPC (Viana Di Prisco and Vertes 2006; Eleore et al. 2011). It is unknown whether ReRh and HPC synapse onto overlapping mPFC cell populations and whether these synapses have divergent receptor expression, temporal properties or are subject to differential neuromodulation and plasticity. Answering these questions is key to understanding how HPC and ReRh inputs are assimilated in mPFC.

Here, we use an optogenetic strategy to detail the physiological properties of ReRh synapses to pyramidal neurons and compare these with HPC inputs to pyramidal neurons in ex vivo mPFC brain slices. We show that ReRh and HPC synapses converge on to the majority of L5 pyramidal neurons and demonstrate marked differences in short-term plasticity between ReRh and HPC and explore their regulation by neuromodulators acetylcholine (ACh) and dopamine, both of which have prominent roles in mPFC physiology. Based on the connectivity of the HPC, ReRh, and mPFC a simple circuit would envisage HPC driving mPFC directly but also driving mPFC indirectly with a delay via ReRh. Alternatively, ReRh can drive mPFC directly but also drive mPFC indirectly with a delay via HPC. Since synaptic plasticity in mPFC is considered important for learning (Meunier et al. 2017; Sabec et al. 2018) but little is known about cooperativity of inputs to mPFC, we finally demonstrate that patterned HPC and ReRh synaptic activity in which HPC leads ReRh (but not in the opposite direction) can interact to induce associative, NMDA receptor-dependent long-term plasticity. This unidirectional cooperativity has important implications for encoding of information within mPFC.

## Materials and Methods

### Animals

All experiments were carried out in naïve male Lister Hooded rats (Envigo) weighing 300–450 g at the start of experiments, a total of 152 animals was used in this study. Animals were housed in groups of 2–4 under a 12 h/12 h light/dark cycle with lights on 20:00–08:00 and were given ad libitum access to food and water. Sacrifice for ex-vivo slices occurred 2–3 h into the dark cycle. All animal procedures were conducted in accordance with the United Kingdom Animals Scientific Procedures Act (1986) and associated guidelines. All efforts were made to minimize suffering and number of animals used.

### Viral Injections

**Figure 1:**
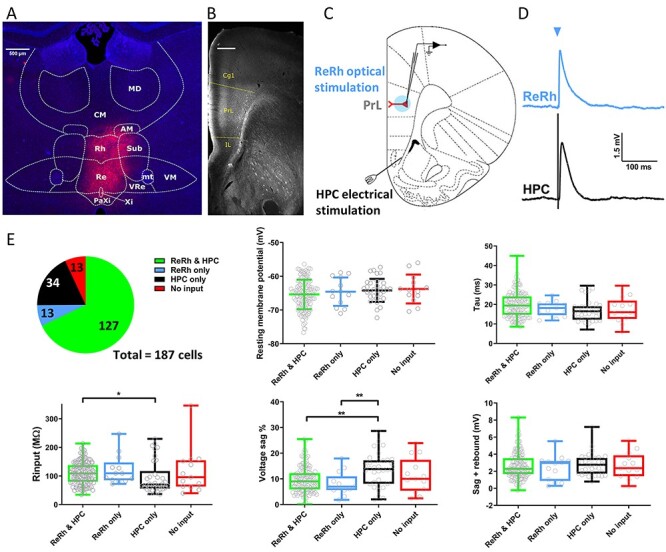
Electrophysiological characterization of L5 pyramidal cells receiving optogenetically activated nucleus reuniens/rhomboid synapses. (*A*) Representative widefield-fluorescence image showing neuronal transduction of nucleus reuniens (Re) and rhomboid nucleus (Rh) following injection of AAV9:CaMKii:hChR2 (E123T/T159C):mCherry (red) and DAPI (blue). VRe, ventral reuniens; Xi, xiphoid; PaXi, paraxiphoid; CM, central medial; AM, anteromedial; VM, ventromedial; MD, mediodorsal; Sub, submedius thalamic nuclei; mt, mammillothalamic tract. (*B*) Monochrome image of mCherry positive fibers in PFC following AAV injection into ReRh. Dotted lines denote the boundaries of prelimbic cortex. mCherry signal is amplified with anti-mCherry antibody. Cg1, cingulate cortex; IL, infralimbic cortex; PrL, prelimbic cortex. Scale bar = 500 μm. (*C*) Schematic of acute mPFC slice with whole-cell recording from layer 5 pyramidal neuron in PrL, light activation of soma and proximal dendrites via microscope objective (blue) and stimulation of hippocampal fiber bundle using conventional stimulating electrode. (*D*) Representative ReRh (blue) and HPC (black) EPSPs. Blue arrow denotes light activation. (*E*) Proportion of cells receiving different permutations of ReRh and HPC inputs, 187 cells from 65 animals. Passive membrane properties measured from −100 pA current injection split by synaptic input. RMP plotted as mean ± standard deviation, one-way ANOVA *F*_(3,183)_ = 1.2, *P* = 0.32. Other parameters one or more column failed Shapiro–Wilk test for normality, box plots show median and interquartile range, whiskers max and min data points. Kruskal–Wallis test *P* values: Tau = 0.074, Rinput = 0.031, Sag % = 0.0036, sag + rebound = 0.84. ^*^/^*^^*^ = *P* < 0.05/0.01 Dunn’s multiple comparisons post-hoc.

Optogenetic transduction of neurons was achieved using AAV9-CaMKii-hChR2 (E123T/T159C)-mCherry (Addgene 35 512; 3.3 × 10^13^ genome copies/mL) obtained from University of Pennsylvania Vector Core or Addgene. The viral vector was chosen due to: 1) the AAV9 serotype having previously been described as having few deleterious effects on synaptic release properties (Jackman et al. 2014); 2) suitability of the CaMKii promotor for transduction of excitatory neurons (Bokor 2002); and 3) the hChR2 (E123T/T159C) “ChETA_TC_” channelrhodopsin variant having a combination of fast kinetics and large photocurrents suitable for activation of distal sites (Berndt et al. 2011; Mattis et al. 2011). Each rat was anesthetized with isoflurane (4% induction and 2.5–3.5% maintenance) and secured in a stereotaxic frame with the incisor bar set 3.3-mm below the interaural line. For these experiments we centered injections at the nucleus reuniens (NRe); bilateral burr holes were made in the skull at the following coordinates with respect to bregma: anterior–posterior (AP)—2.0 mm, mediolateral (ML) **±** 1.4 mm. Virus was front loaded into a 33-gauge 12° beveled needle (Esslab) attached to a 5-μL Hamilton syringe which was mounted at a 10° angle in the ML plane to avoid the sinus, with the eyelet of the needle facing medially. The needle was lowered 7.5-mm below the surface of the skull measured from the burr hole and 100 nL of virus was delivered via each burr hole at a rate of 200 nL.min^−1^, with the needle left in situ for 10 min after each injection. Viral injections transduced neurons approximately ±1.0 mm in the anteroposterior axis and produced strong mCherry expression in NRe, rhomboid nucleus, and xiphoid with sparse expression in adjacent ventral reuniens (also referred to as the perireuniens), paraxiphoid, and submedius thalamic nuclei (Fig. 1*A*). Of those nuclei, only the reuniens, ventral reuniens, and rhomboid nuclei project to prelimbic cortex (Hoover and Vertes 2007; Alcaraz et al. 2015; Salay et al. 2018), these are hereafter referred to collectively as ReRh. Cell body labelling was infrequently observed in mPFC L6, which was presumed to be retrograde transduction, however there was no evidence for retrograde transduction of L5 pyramidal neurons (e.g., short-latency, glutamate antagonist-insensitive cation currents, or mCherry labelling). For HPC viral injections, coordinates were AP −6.3 mm, ML **±** 5.5 mm, dorsoventral −5.8 mm, measured from bregma. Hippocampal viral injection volume was 500 nL per hemisphere.

### Acute Slice Preparation

After a minimum of 10 days following viral injection, animals were anesthetized with 4% isoflurane and decapitated. Brains were rapidly removed and placed into ice-cold sucrose solution (in mM: 189 sucrose, 26 NaHCO_3_, 10 d-glucose, 5 MgSO_4_, 3 KCl, 1.25 NaH_2_PO_4_, and 0.2 CaCl_2_) bubbled with 95% O_2_/5% CO_2_. The brain was sectioned at 11° using a custom brain matrix as previously described (Banks et al. 2015) and modified coronal slices were cut at 350-μm thickness using a vibratome (7000smz-2, Camden Instruments), hemisected and incubated at 34 °C for 1 h after dissection in a slice holding chamber filled with artificial cerebrospinal fluid (aCSF, in mM: 124 NaCl, 26 NaHCO_3_, 10 d-glucose, 3 KCl, 2 CaCl_2_, 1.25 NaH_2_PO_4_, and 1 MgSO_4_). Slices were subsequently stored at room temperature until use.

### Electrophysiology

Slices at ~ 3.0-mm anterior to bregma were used for electrophysiology, placed in a submerged recording chamber and perfused with 34 °C aCSF at ~ 2 mL min^−1^. A stimulating electrode (FH-Co, CBABAP50) was placed on the hippocampal fiber bundle as previously described (Banks et al. 2015). Pyramidal neurons in prelimbic cortex (layer 5 unless otherwise stated) were targeted under oblique infra-red illumination based on somatic morphology and patch clamped using 2–6 MΩ boroscillicate glass electrodes (GC150-10F, Harvard Apparatus) filled with potassium gluconate internal for current-clamp experiments (in mM: 120 k-gluconate, 40 HEPES, 10 KCl, 2 NaCl, 2 MgATP, 1 MgCl, 0.3 NaGTP, 0.2 EGTA, and 0.1 Alexa-594 hydrazide) or cesium methylsulfonate for voltage clamp (130 CsMeSO_4_, 10 HEPES, 8 NaCl, 5 QX-314Cl, 4 MgATP, 0.5 EGTA, 0.3 NaGTP, and 0.1 Alexa-594 hydrazide). Wide-field fluorescence was used at the end of experiments to confirm pyramidal cell morphology (prominent apical dendrite extending toward layer 1). Recordings were obtained using a Molecular Devices Multiclamp 700A or 700B, filtered at 4 KHz and digitized at a sample frequency ≥ 20 KHz with WinLTP2.30 (Anderson and Collingridge 2007) or pClamp10 software.

For current clamp recordings resting membrane potential (RMP) was recorded immediately after entering the whole-cell configuration. Intrinsic membrane properties were recorded as previously described (Barker et al. 2017). Neurons were kept at −70 mV by injection of constant current throughout experiments. Basal synaptic stimulation occurred every 10 s, alternating between optogenetic stimulation of ReRh and electrical stimulation of HPC input to achieve a 0.05-Hz intra-pathway basal stimulation frequency. Stimulation at this frequency was continued until responses in both pathways were stable for 10 min before application of pharmacological agents, or for 5 min for induction of activity-dependent plasticity, cells were then recorded for 40 min after these manipulations to monitor long-term effects. To examine effects on short-term plasticity, 5 and 10-Hz transmission was examined before, and then at least 10 min following bath application of pharmacological agents. Only neurons in which both pathways were measurable were used for synaptic experiments, this constituted 68% of neurons. Optogenetic stimulation was applied with a 470-nm LED (M470L3, Thorlabs) triggered by TTL pulses sent to an LEDD1B driver (Thorlabs) directed onto the soma and proximal dendrites via a ×40 immersion objective (Olympus LUMPLFLN40XW) resulting in a 660-μm diameter illumination. The LED was usually driven at maximal strength, resulting in 4.35 mW.mm^−2^ light density. EPSP amplitude was adjusted by changing the light pulse duration (0.2–5 ms, typically 1 ms). The hippocampal fiber bundle was stimulated (0.1-ms duration) using a bipolar concentric stimulation electrode (CBAPB125, FHC) and Digitimer DS3 constant current stimulator. In some experiments, as detailed in the results, HPC inputs were light stimulated in ex vivo slices following prior in vivo HPC viral transduction of ChETA_TC_. Stimulation intensity of each pathway was adjusted to achieve subthreshold EPSPs that were typically 2–8 mV.

NMDAR: AMPAR ratios were obtained by recording AMPAR-mediated synaptic potentials at −70 mV and, following bath application of 5-μM NBQX and 50-μM picrotoxin, NMDARs at +40 mV. Ro 25-6981 experiments were conducted at −40 mV to minimize cell death during experiments. NMDA receptor weighted decay time constants (τ_w_) were calculated in pClamp by fitting a double exponential curve between 90 and 10% of +40 mV NMDAR responses using the formula τ_w_ = τ1^*^A1/(A1 + A2) + τ2^*^A2/(A1 + A2) (Matta et al. 2011). For MK-801 experiments, isolated NMDA receptor currents were obtained at +40 mV, 40 μM (+)MK-801 malleate was then bath applied for 10 min in the absence of stimulation with the cell held at −70 mV. Cells were then held at +40 mV again before resumption of stimulation at 0.1 Hz. For pairing plasticity experiments a baseline of 5 min was achieved within 10 min of break-in or cells were discarded to prevent washout (Malinow and Tsien 1990), intrinsic membrane properties were thus not recorded in those experiments.

Pharmacological agents were made up as stock solutions, stored at −20 °C, and diluted prior to bath application at concentrations and for the durations described in the results. These were purchased from Tocris Bioscience or Hello Bio.

### Staining and Imaging

Rats were anesthetized with Euthatal and perfused transcardially with phosphate buffer (PB) followed by 4% paraformaldehyde (PFA). Brains were removed and postfixed in 4% PFA for 2 h before being transferred to 30% sucrose in PB for 48 h. Coronal sections (40 μm) were cut on a cryostat. Sections were washed 3 times in phosphate buffered saline (PBS), incubated for 30 min in 1% H_2_O_2_ in PBS, washed 3 times in 0.2% Triton X in PBS (PBS-T), incubated for 1 h in blocking solution (5% normal goat serum, 2.5% BSA, in PBS-T), and then incubated in primary antibody solution (anti-mCherry rabbit polyclonal [Abcam ab167453]; 1:1000 in blocking solution) for 24 h at RT. Sections were then washed 4 times in PBS-T, incubated in secondary antibody solution (goat anti-rabbit conjugated to Alexa Fluor 594 [Abcam ab150080]; 1:500 in blocking solution) for 2 h at RT and then washed a further 4 times in PBS-T, mounted and coverslipped with Vectashield with DAPI (Vector Laboratories H-1500) mounting medium. Images were acquired using a widefield fluorescence microscope (Leica).

### Analysis and Statistics

Synaptic responses were averaged into 1-min bins and analyzed using WinLTP2.3 or pClamp10. Intrinsic membrane properties were imported to MATLAB using SourceForge and analyzed using code supplied by Dr Jon Brown (Exeter University) as described previously (Barker et al. 2017). Experiments used *N* numbers typical of the field. With the exception of data in Fig. 1*E* and Supplementary Fig. S1*A*–*C*, we typically replicated experiments in one cell per animal, number of cells was thus considered as experimental *N* for statistical purpose, however numbers of both cells and animals used in each experiment are reported. Data are expressed as mean ± standard error of the mean (SEM) unless otherwise stated. Statistical tests were done using SPSS (IBM) or Graphpad Prism 7. Data sets were tested for normality prior to analysis using Shapiro–Wilk test to determine use of parametric or nonparametric tests as follows: unpaired observations were compared using student’s *t*-test or nonparametric Mann–Whitney *U* (2-tailed), unpaired observations of more than 2 groups were made using one-way analysis of variance (ANOVA) or nonparametric Kruskal–Wallis with Tukey’s or Dunn’s post-hoc comparisons, respectively. Paired comparisons of 2 groups were made with paired *t*-test or Wilcoxon matched-pair signed rank (2-tailed). Comparisons across a range of within- and between-subjects variables were made with 2-way repeated-measures ANOVA with Sidak’s post-hoc multiple comparisons. Short-term plasticity was assessed from normalized peak amplitudes with repeated-measures ANOVA with Greenhouse–Geisser corrections applied where Maulchy’s test for Sphericity significance was < 0.05. For pairing experiments, plasticity in individual pathways was assessed by comparison of average EPSP amplitudes during baseline and 30–40-min post-pairing (follow-up). For all experiments, significance was reported at *P* < 0.05.

## Results

### Characterizing L5 Pyramidal Neurons in Prelimbic Cortex That Receive Reuniens/Rhomboid Nuclei and Hippocampal Inputs

Of the thalamic nuclei with projections to mPFC, viral injections into ventral midline thalamus transduced reuniens, ventral reuniens and rhomboid nuclei (from now on collectively referred to as ReRh) with channelrhodopsin variant ChETA_TC_ (AAV9-CaMKii-hChR2 (E123T/T159C)-mCherry; Fig. 1*A*) resulting in mCherry labelling (Fig. 1*B*) across all layers (1–6) of mPFC (Vertes et al. 2006). In this study, we focused on the ReRh and HPC projections to layer 5 pyramidal neurons—one of the primary output cells of prelimbic cortex and the main target for HPC afferents (Liu and Carter 2018). Modified coronal mPFC slices were prepared as described previously (Parent et al. 2010; Banks et al. 2015). To activate ReRh axons in PFC an LED was directed over L5 cell soma and proximal dendrites via the microscope objective (Fig. 1*C*). To activate the HPC fiber bundle, a stimulating electrode was positioned between the dorsal tenia tecta and the nucleus accumbens (Fig. 1*C*). The hippocampal origin of fibers in this region, in slices cut as described in the methods, has been demonstrated using anterograde tracers (Parent et al. 2010). Crucially, we have previously shown that HPC lesions almost entirely abolish EPSCs resulting from electrical stimulation in this region in layer V pyramidal neurons, demonstrating that the majority of these fibers are hippocampal in origin (Banks et al. 2015). Therefore, combining these approaches allowed us to simultaneously compare light evoked ReRh and electrically evoked HPC inputs onto the same cells in mPFC.

ChETA_TC_ activation of ReRh afferents resulted in EPSPs with simple waveforms, providing direct evidence that neurons of ReRh synapse upon L5 pyramidal cells in mPFC (Fig. 1*D*). We found that there is a high degree of convergence of HPC and ReRh pathways onto individual mPFC pyramidal cells; 68% of L5 pyramidal neurons received input from both ReRh and HPC, 18% from HPC alone, and 7% from ReRh alone (Fig. 1*E*).

Layer 5 pyramidal neurons can be separated into 2 simplified subtypes based on their projection targets: intratelencephalic (IT) which principally project cortically, and pyramidal tract (PT) which principally project subcortically. These classes of neuron have distinct electrophysiological properties, most notably PT neurons show pronounced Ih (Dembrow et al. 2010). Furthermore, long-range inputs to mPFC, including those from HPC, have shown preferential targeting of layer 5 pyramidal neuron subtypes (Dembrow et al. 2015; Cheriyan et al. 2016; Anastasiades et al. 2018; Liu and Carter 2018). Therefore, to investigate whether HPC or ReRh afferents differentially target IT or PT neurons we compared intrinsic passive and active membrane properties of cells receiving input from both ReRh and HPC, from either pathway alone, or from neither input. Cells that only had input from HPC had significantly lower median input resistance compared with cells which received both ReRh and HPC input (Fig. 1*E*). Cells with only HPC input also expressed the largest percentage of I_h_-mediated voltage sag; however there was no difference in the sum of absolute level of sag and the rebound from hyperpolarization, a measure which has been used to classify cortically and subcortically projecting cells previously (Gee et al. 2012; Lee et al. 2014). RMP and membrane time constant were the same across all input groups (Fig. 1*E*).

Regarding active membrane properties, action-potential threshold of cells receiving only HPC input was more hyperpolarised than that of either group of neurons which received ReRh input (see Supplementary Fig. S1*A*). No other difference in action potential properties (see Supplementary Fig. S1*A*), number of spikes or spike frequency adaptation was found between groups (see Supplementary Fig. S1*B*). Amplitudes of afterhyperpolarisation and afterdepolarisation were not different across groups (see Supplementary Fig. S1*C*).

These results show that cells receiving only HPC input have lower input resistance and show greater degree of sag (more closely resembling PT cells) than those receiving both ReRh and HPC inputs, or ReRh alone. However, it should be noted that the largest proportion of cells receiving HPC input in this study are represented by the HPC/ReRh group, among which there is a large degree of variance in intrinsic electrophysiological properties and therefore likely contains both IT and PT like cells. All subsequent experiments comparing properties of HPC and ReRh inputs were performed only in cells with both ReRh and HPC inputs.

### Comparison of Reuniens/Rhomboid Nuclei and Hippocampal Synaptic Properties

**Figure 2:**
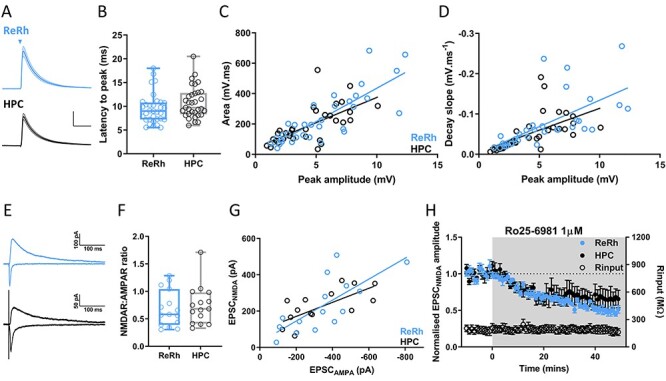
ReRh and HPC synapses are indistinguishable. (*A*) Average waveforms of ReRh and HPC EPSPs, stimulation denoted by triangle, electrical stimulation artifacts removed for clarity. Traces show mean ± SEM waveform, scale bars = 2 mV/50 ms. (*B*) Latency from stimulation to EPSP peak, box plot shows median, 25th and 75th percentiles, whiskers maxima and minima. Individual values shown as open circles. (Mann–Whitney test, *U* = 407, *P* = 0.077, *n* = 33 cells from 28 animals). (*C*) EPSP peak amplitude versus area of ReRh and HPC EPSPs. Linear regression slopes were not significantly different (*F*_(1,62)_ = 1.5, *P* = 0.23). (*D*) EPSP peak amplitude versus decay slope from 90% to 15% of EPSP peak. Linear regression slopes were not significantly different (*F*_(1,62)_ = 0.3, *P* = 0.57). (*E*) Representative −70-mV AMPAR-mediated (negative-going) and +40-mV NMDAR-mediated (positive-going) EPSCs resulting from ReRh (blue) and HPC (black) stimulation. (*F*) NMDAR:AMPAR ratios of ReRh and HPC EPSCs were not significantly different (Mann–Whitney *U* = 99, *P* = 0.60, *n* = 15 cells from 10 animals). (*G*) EPSC_AMPA_ versus EPSC_NMDA_ for ReRh and HPC inputs. Linear regression slopes were not significantly different (*F*_(1,26)_ = 1.05, *P* = 0.32). (*H*) EPSC_NMDA_ inhibition by bath application of GluN2B selective antagonist Ro25–6981 (3 μM) as indicated by gray shaded region. Mann–Whitney *U* = 12, *P* = 0.4. *N* = 6 cells from 5 animals.

Activation of ReRh and HPC afferents resulted in simple waveform EPSPs which were highly alike (Fig 2*A*). Bath application of tetrodotoxin (TTX, 0.5 μM) abolished ReRh (and HPC) EPSPs (see Supplementary Fig. S1*D*, *E*) confirming that ChETA_TC_-evoked ReRh responses are action potential dependent. Addition of voltage-dependent K^+^ blocker 4-AP (100 μM) did not result in ReRh transmission; EPSPs were only partially restored when stimulus duration was increased (see Supplementary Fig. S1*D*, *E*). These data demonstrate that opsin stimulation results in ReRh EPSPs onto L5 pyramidal neurons that are monosynaptic and action potential dependent (Petreanu et al. 2009). In further support of the monosynaptic nature of ReRh EPSPs under control conditions, ChETA_TC_-EPSPs were of short latency and not significantly different to those evoked electrically from HPC fibers (Fig. 2*B*, Mann–Whitney *P* = 0.077).

Bath application of ionotropic glutamate receptor antagonists NBQX and D-AP5 completely blocked both ReRh (see Supplementary Fig. S1*D*, *E*) and HPC responses (see Supplementary Fig. S1*F*), confirming that ReRh input is glutamatergic (Hur and Zaborszky 2005). Linear regression of EPSP amplitude versus area for ReRh and HPC yielded equal slopes (Fig. 2*C*, *P* = 0.23) and regression of EPSP amplitude versus decay slope was also indistinguishable (Fig. 2*D*, *P* = 0.57). Since ReRh and HPC EPSPs are recorded within the same cells, there can be no difference in intrinsic electrical properties between cells receiving these synaptic pathways. This suggests that there is little difference in AMPA receptor subunit composition between synapses, and that the dendritic location of activated synapses is similar (Magee 2000).

NMDA receptors, in particular GluN2B subunit containing receptors, play a key role in supporting persistent firing, which is thought to contribute to working memory in PFC (Monaco et al. 2015), moreover expression of GluN2B subunits may be input specific (Flores-Barrera et al. 2014). We therefore examined NMDA receptor expression and stoichiometry at ReRh and HPC synapses. No difference in the ratio of NMDAR: AMPAR EPSCs was seen (Fig. 2*E*, *F*; Mann–Whitney, *P* = 0.60) and linear regression of EPSC_AMPA_ versus EPSC_NMDA_ slopes were equal (Fig. 2*G*, *P* = 0.32). Weighted decay time constants were not significantly different (Tw: ReRh = 135.3 ± 44.3 ms, HPC = 128.9 ± 32.8 ms, and Mann–Whitney *U* = 34, *P* = 0.9) and inhibition of GluN2B subunit-containing receptors by Ro 25-6981 (3 μM) was indistinguishable between the 2 inputs (Fig. 2*H*, Mann–Whitney, *P* = 0.4), indicating that overall levels of NMDAR expression and stoichiometry are not appreciably different at ReRh and HPC synapses. Altogether these data show that single evoked ReRh and HPC EPSCs in prelimbic cortex L5 pyramidal cells are indistinguishable and likely to have similar postsynaptic ionotropic glutamate receptor composition.

### NRe/Rhomboid, but Not HPC, Afferents to mPFC Undergo Short-Term Depression at Theta Frequency

**Figure 3:**
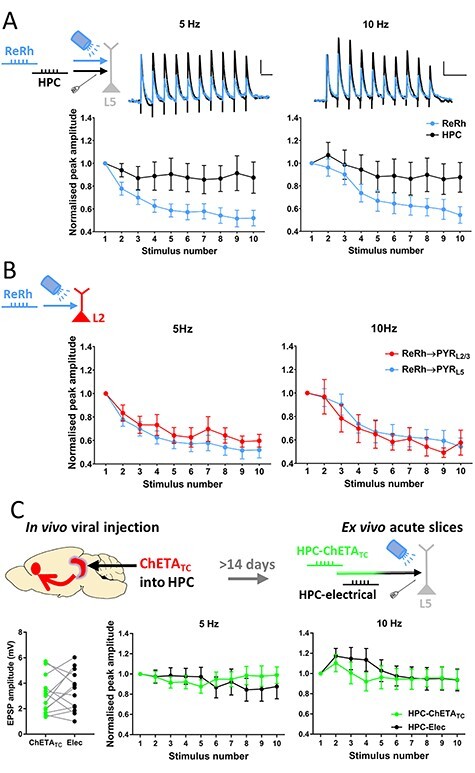
Reuniens/rhomboid inputs to prelimbic cortex depress at theta frequency. (*A*) ReRh inputs to L5 pyramidal neurons undergo strong short-term depression, and show different plasticity pattern to HPC inputs at 5 Hz (repeated measures 2-way ANOVA: main effect of pathway *F*_(1,11)_ = 8.5, *P* = 0.014; main effect of response number *F*_(1.9,20.6)_ = 5.1; *P* = 0.018, interaction *F*_(3.1, 34.1)_ = 4.4, *P* = 0.0095) and 10 Hz (pathway *F*_(1,11)_ = 5.0, *P* = 0.048; response number *F*_(3.2,35.4)_ = 27.9, *P* = 1.1 × 10^−9^; interaction *F*_(4.3,47.0)_ = 8.5, *P* = 0.00002; Greenhouse–Geisser correction applied, both frequencies). *N* = 12 cells from 11 animals. Scale bars = 0.3 mV/200 ms. (*B*) ReRh inputs to L2/3 pyramidal cells show equal degree of short-term depression as inputs to L5 at 5 Hz (Repeated-measures 2-way ANOVA: main effect of layer *F*_(1,19)_ = 0.71, *P* = 0.41; main effect of response number *F*_(8,61.0)_ = 15.3, *P* = 2.6 × 10^−16^; interaction *F*_(3.2,61)_ = 0.45, *P* = 0.73) and 10 Hz (main effect of layer: *F*_(1,19)_ = 0.12, *P* = 0.73; main effect of response number *F*_(8,62.7)_ = 30.3, *P* = 6.8 × 10^−28^; interaction *F*_(3.3,62.7)_ = 0.9, *P* = 0.44; Greenhouse–Geisser correction applied, both frequencies. L2/3 *n* = 9 cells from 5 animals; L5 data repeated from Fig 3*A*). (*C*) Following injection of AAV9-CaMKii-hChETA_TC_-mCherry into intermediate/ventral HPC, acute mPFC slices were made and HPC-mPFC transmission evoked by electrical and optogenetic stimulation were compared. EPSPs evoked by ChETA_TC_ and electrical stimulation were of similar amplitude (paired *t*-test, *t*_(12)_ = 0.78, *P* = 0.45). No difference in short-term plasticity was observed at 5 (main effect of stimulation method 5 Hz: *F*_(1,12)_ = 0.07, *P* = 0.79; main effect of response number *F*_(2.1,30.0)_ = 0.45, *P* = 0.65; interaction *F*_(3.1,36.9)_ = 2.2, *P* = 0.10) or 10 Hz stimulation frequency (stimulation method *F*_(1,12)_ = 0.38, *P* = 0.55; response number *F*_(2.1,24.7)_ = 4.6, *P* = 0.02, interaction *F*_(2.6,31.7)_ = 1.1, *P* = 0.38). Greenhouse–Geisser corrections applied. *N* = 13 cells from 5 animals.

Next we examined short-term plasticity of ReRh and HPC inputs to L5 pyramidal neurons at frequencies relevant to ReRh-mPFC and HPC-mPFC theta (4–12 Hz) oscillations (Jones and Wilson 2005; Siapas et al. 2005; O'Neill et al. 2013; Spellman et al. 2015; Hallock et al. 2016; Roy et al. 2017) and the tonic firing frequency of NRe matrix cells (Walsh et al. 2017). In contrast to in vivo recordings showing paired-pulse facilitation (Viana Di Prisco and Vertes 2006), ReRh EPSPs strongly depressed when activated at both 5 and 10 Hz (Fig. 3*A*), an effect we did not see for HPC EPSPs (Liu and Carter 2018). Pronounced short-term depression of ReRh responses was also observed in L2/3 pyramidal cells, with no difference in depression seen between layers 2/3 and 5 (Fig. 3*B*). These data demonstrate a functional projection from ReRh to L2/3 pyramidal neurons and show that, in contrast to the HPC projection, ReRh projections to mPFC pyramidal cells undergo short-term depression irrespective of mPFC lamina.

We first considered the possibility that short-term depression of ReRh inputs was an artifact of optogenetic activation since ChR2 variants might be unable to evoke spiking reliably at higher frequencies (Berndt et al. 2011) and transduction of neurons with AAVs itself may affect synaptic release (Jackman et al. 2014). To test this possibility, we instead transduced the HPC with ChETA_TC_ and then compared, in ex vivo slices, HPC EPSPs evoked onto the same cell by electrical and optical stimulation. Electrical and ChETA_TC_ evoked HPC EPSPs were of similar initial amplitude and no significant difference was observed between optical and electrical stimulation of HPC afferents at theta frequencies (Fig. 3*C*), thereby demonstrating the ability of ChETA_TC_ to evoke high fidelity transmission at 5 and 10 Hz and strongly suggesting that ReRh short-term depression is not due opsin desensitization. We also compared the degree of short-term depression observed in ReRh inputs to the duration of light pulses used to evoke ReRh EPSPs, with no relationship observed between these 2 variables (see Supplementary Fig. S2*A*). These data demonstrate that short-term depression of ReRh transmission is not an artifact of optogenetic activation of ReRh inputs, due to, for example, presynaptic calcium influx through the opsin. It should be noted that it is not possible in vitro to selectively stimulate axons ReRh into mPFC electrically, therefore the electrical versus opsin stimulation control experiments cannot be performed for ReRh.

For completeness we also tested in hippocampal afferents the range of stimulation frequency possible with ChETA_TC_. At frequencies of 20 Hz and above we saw deficits in optically-evoked HPC transmission compared with electrically evoked transmission (see Supplementary Fig. S2*B*), with optical EPSPs showing attenuated amplitude at 20 Hz and an inability to evoke subsequent EPSPs at 100 Hz, possibly owing to failure to recover from desensitization between pulses.

**Figure 4:**
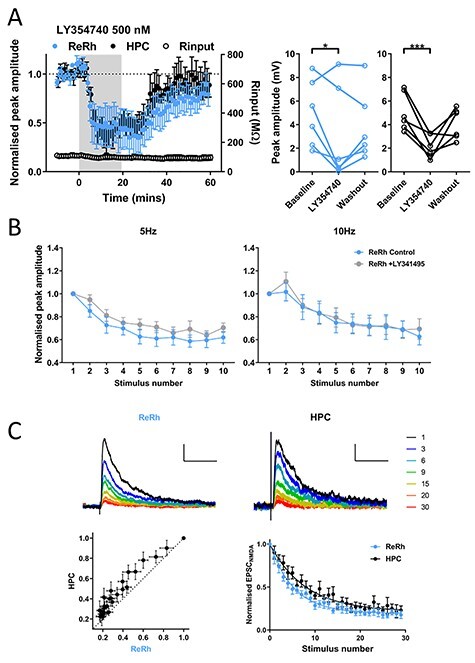
ReRh and HPC are inhibited by group II mGluR activation, but high probability of release underlies short-term depression of ReRh inputs. (*A*) Activation of group II mGluRs with LY354740 (500 nM) reveals no difference in acute depression of ReRh and HPC inputs measured during the final 10 min of drug application (2-way repeated measures ANOVA: main effect of timepoint *F*_(2,20)_ = 13.3, *P* = 0.0002; pathway *F*_(1,10)_ = 0.02, *P* = 0.90; interaction *F*_(2,20)_ = 0.81, *P* = 0.46; Sidak’s post-hoc comparisons shows difference vs. baseline, ReRh *P* = 0.024, HPC *P* = 0.0009). *N* = 7 cells from 7 animals. (*B*) LY341495 (100 μM) did not affect short-term plasticity of ReRh input at 5 or 10 Hz (RM ANOVA; 5 Hz: main effect of drug *F*_(1,8)_ = 4.8, *P* = 0.059; main effect of response number *F*_(4.0,32.3)_ = 23.6, *P* = 3 × 10^−9^; interaction *F*_(4.4,35.2)_ = 0.85, *P* = 0.51; 10 Hz: main effect of drug *F*_(1,8)_ = 0.47, *P* = 0.51; response number *F*_(2.6,20.7)_ = 28.8, *P* = 2.9 × 10^−7^; interaction *F*_(2.5,20.2)_ = 2.6, *P* = 0.089). Greenhouse–Geisser corrections applied. *N* = 9 cells from 6 animals. (*C*) Activity-dependent block of isolated NMDA EPSCs by MK-801 (40 μM). Example blockade of EPSC_NMDA_ measured at +40 mV in ReRh and HPC pathway, traces colored by stimulus number in presence of MK-801, normalized to amplitude of first response, scale bars: 0.25 of normalized peak/100 ms. Plot of ReRh versus HPC amplitudes shows data lie above the identity line. Decay of ReRh is significantly faster than decay of HPC (single exponential curve constrained to Y0 = 1, ReRh τ = 5.4, HPC τ = 7.9, extra sum of squares *F*-test, *F*_(2,532)_ = 19.0, *P* < 0.0001). *N* = 10 cells from 8 animals.

Having demonstrated that pronounced short-term depression is not an artifact of optogenetics but is a physiological feature of ReRh-mPFC synapses we set about using electrical stimulation of HPC and opsin stimulation of ReRh to further compare these 2 pathways. We first explored possible mechanisms for the depression at ReRh synapses, compared with HPC synapses. One potential mechanism for synaptic depression is activation of presynaptic metabotropic glutamate receptors (mGluRs) leading to autoinhibition. Since activation of group II mGluRs depresses MD inputs to mPFC (Joffe et al. 2020) and mGluR2 has been shown to be strongly expressed in reuniens and rhomboid nuclei (Ohishi et al. 1998; Lourenco Neto et al. 2000), we therefore hypothesized that differential expression of group II mGluRs may underlie the differences in short-term depression occurring at ReRh and HPC inputs. However, application of the Group II mGluR agonist LY354740 (500 nM) resulted in similar levels of acute depression of both ReRh and HPC inputs (Fig. 4*A*). Furthermore, neither the broad spectrum mGluR antagonist LY341495 (100 μM) nor the selective group II mGluR antagonist EGLU (10 μM) affected the short-term dynamics at 5 or 10 Hz in either pathway (Fig. 4*B*; see Supplementary Fig. S3*A*).

We next explored the possibility that recruitment of a G-protein coupled potassium conductance is responsible for ReRh depression. Theta range stimuli were thus delivered before and after application of the GABA_B_ receptor antagonist CGP55845 (1 μM), however no effect was seen on either pathway (see Supplementary Fig. S3*B*). Additionally, neither the NMDAR antagonist D-AP5 (50 μM; see Supplementary Fig. S3*C*) nor the nicotinic receptor antagonist mecamylamine (1 μM; see Supplementary Fig. S3*D*) affected short-term plasticity, suggesting no role for presynaptic NMDARs or nAChRs in regulation of synaptic release.

Given the above results we hypothesized that presynaptic release mechanisms in ReRh synapses most likely explain short-term depression, possibly owing to a high initial release probability as has been shown for other thalamocortical synapses (Gil et al. 1999). To address this question, we measured the rate of blockade of pharmacologically isolated NMDAR-mediated currents (EPSC_NMDA_) by the use-dependent NMDA receptor antagonist MK-801 (Hessler et al. 1993; Gil et al. 1999). As MK-801 blocks only open channel pores, pathways with high release probability are predicted to activate NMDA receptors at a large proportion of their synaptic sites, thus resulting in a faster rate of block by MK-801 compared with a pathway with lower release probability. ReRh EPSC_NMDA_ were blocked faster than HPC EPSC_NMDA_ as shown by plotting EPSC_NMDA_ amplitudes of ReRh and HPC for each trial against each other, with the data points lying above the line of identity (Fig. 4*C*). Furthermore, decay curves of the time course of block by MK-801 were fit by single exponential curves with significantly different parameters (*P* < 0.0001). Together these data show that ReRh has a high probability of release, as observed at other thalamocortical synapses, and this is likely to underlie the marked differences between ReRh and HPC transmission observed during theta-frequency stimulation. In addition, when optogenetic and electrical stimulation of hippocampal afferents were compared we found no difference in MK-801 blockade of EPSCs between the 2 stimulation conditions (see Supplementary Fig. S4*A*, *B*). These data show that under the conditions of our experiments, optogenetic stimulation does not alter probability of release and further confirms the physiological differences between ReRh and HPC synapses onto L5 pyramidal neurons in mPFC.

### Cholinergic Neuromodulation of HPC, but Not ReRh Afferents, via M2 Muscarinic Receptors

**Figure 5:**
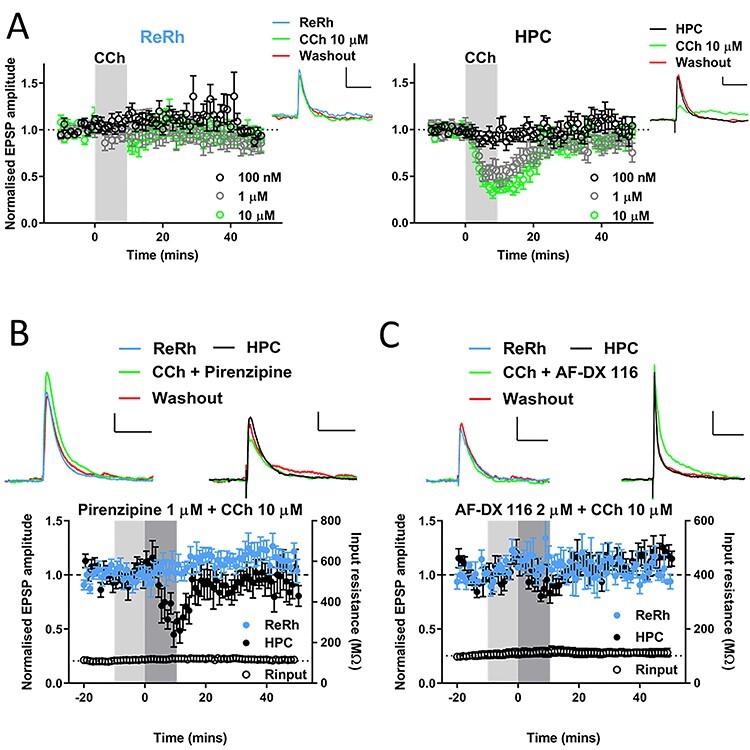
Cholinergic modulation of hippocampal, but not nucleus reuniens/rhomboid inputs to prelimbic cortex via M2 muscarinic receptors. (*A*) Pooled data showing 10-min bath application of cholinergic agonist carbachol (shaded region) at different concentrations has no effect upon ReRh (left) input to PrL, but reversibly depresses HPC inputs (right) in a concentration-dependent manner. Example EPSPs for each pathway at baseline (−10 to −1 min), acute (10–19 min) and washout (40–49 min) shown from a representative 10 μM experiment (scale bars = 1 mV, 100 ms; *n* = cells/animals: 100 nM = 9/8; 1 μM = 6/4; 10 μM = 8/7). (*B*) Selective M1 muscarinic antagonist pirenzipine does not block depression of HPC by 10-μM CCh. Pirenzipine (1 μM) pre-applied during light shaded region and co-applied with CCh during dark shaded region. EPSPs from a representative cell are shown above pooled data, scale bars = 1 mV/100 ms. *N* = 7 cells from 6 animals. (*C*) Selective M2 muscarinic antagonist AF-DX 116 blocks depression of HPC by 10-μM CCh. AF-DX 116 (2 μM) pre-applied during light shaded region and co-applied with CCh during dark shaded region. EPSPs from a representative cell are shown above pooled data, scale bars = 1 mV/100 ms for ReRh and 2 mV/100 ms HPC. *N* = 5 cells from 5 animals. For plots of individual experiments and statistics please refer to Supplementary Fig. S5.

Acetylcholine signaling in mPFC is essential for associative recognition memory (Barker and Warburton 2009) and it is likely that ReRh inputs to mPFC are active during phasic ACh release in mPFC, however it is unknown whether ReRh afferents are sensitive to modulation by ACh. To address this question, we bath applied the broad-spectrum cholinergic agonist carbachol (CCh) for 10 min. To our surprise, ReRh afferent input to L5 pyramidal neurons was unaffected by CCh (Fig. 5*A*, see Supplementary Fig. S5*A*). In contrast, low micromolar concentrations of CCh produced a strong, reversible attenuation of HPC inputs (Fig. 5*A*, individual experiments shown in see Supplementary Fig. S5*A*; 2-way repeated measures ANOVA pathway × concentration × timepoint interaction of CCh effects (*F*_(4,40)_ = 3.0, *P* = 0.029). In addition, there was no difference in the effects of CCh when comparing optogenetic stimulation or electrical stimulation of HPC afferents recorded in the same cells (see Supplementary Fig. S4*C*–*E*). These data show that optogenetic stimulation does not alter properties of synaptic transmission and further demonstrate the pathway specificity of electrical HPC stimulation.

We next sought to characterize the receptors responsible for the depression of HPC afferents by CCh, focusing on muscarinic receptors as these have previously been shown to mediate CCh depression of various synapses in mPFC (Wang and Yuan 2009; Huang and Hsu 2010; Caruana et al. 2011). The M1 selective muscarinic antagonist pirenzepine (1 μM) did not prevent the HPC-mPFC CCh depression (Fig. 5*B*, see Supplementary Fig. S4*B*). In contrast, the M2 receptor antagonist AF-DX 116 (2 μM) completely blocked the acute depression of HPC responses by CCh (*P* = 0.001; Fig. 5*C*, see Supplementary Fig. S5*B*). ReRh inputs were not significantly modulated by CCh in the presence of M1 or M2 selective antagonists, thus ruling out the possibility that M1 and M2 receptor activation have equal and opposite effects that mask each other (Fig. 5*B*, *C*, see Supplementary Fig. S5*B*). Together these data suggest that during increased cholinergic tone, HPC inputs to L5 pyramidal cells in mPFC are selectively inhibited via M2 muscarinic receptors. In contrast the ReRh–mPFC input, somewhat uniquely among studied mPFC glutamatergic synapses, is not strongly modulated by cholinergic activation.

### Dopaminergic Neuromodulation of ReRh and HPC Afferents

Having shown that ReRh and HPC inputs are differentially controlled by cholinergic neuromodulation, we wanted to determine whether input specific modulation was a common feature of these pathways. Dopaminergic signaling in the mPFC plays a key role in executive function in mPFC (Ott and Nieder 2019) and modulates synaptic transmission in mPFC (Law-Tho et al. 1994; Gao et al. 2001; Seamans et al. 2001; Urban et al. 2002; Gonzalez-Islas and Hablitz 2003). We therefore examined the effect of D1R and D2R dopamine receptor subclass agonists on basal ReRh and HPC EPSPs.

**Figure 6:**
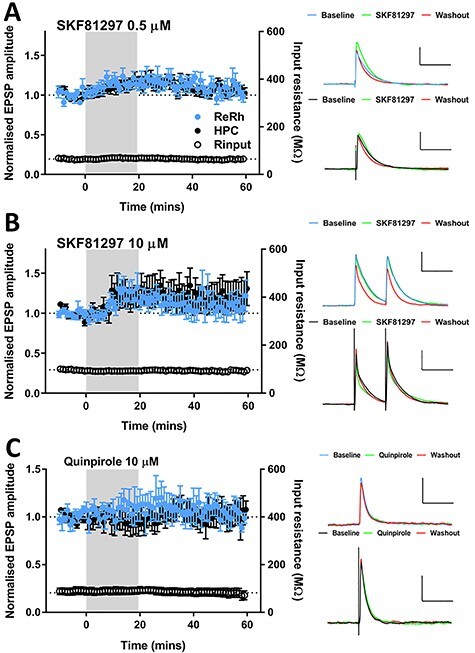
Dopaminergic modulation of basal ReRh and HPC inputs to mPFC. (*A*) D1R-like dopamine agonist SKF81297 bath applied at 0.5 μM caused a modest, reversible increase in transmission which was not pathway specific (2-way ANOVA, main effect of timepoint: *F*_(2,48)_ = 5.4, *P* = 0.0078; pathway *F*_(1,24)_ = 0.4, *P* = 0.54; interaction *F*_(2,48)_ = 1.8, *P* = 0.17). Values = mean ± SEM. Representative EPSPs at baseline, final 10 mins of drug application and final 10 min of recording, scale bars = 3 mV/100 ms. *N* = 13 cells from 13 animals. (*B*) SKF81297 at 10 μM did not result in a significant alteration of ReRh or HPC EPSPs (2-way ANOVA, main effect of timepoint *F*_(2,64)_ = 1.1, *P* = 0.34; main effect of pathway *F*_(1,32)_ = 0.07, *P* = 0.8, interaction *F*_(2,64)_ = 0.34, *P* = 0.71). Values = mean ± SEM. Representative EPSPs shown, scale bars = 3 mV/100 ms. *N* = 18 cells from 16 animals. (*C*) D2R-like dopamine agonist quinpirole (10 μM) does not affect basal ReRh or HPC transmission (2-way ANOVA, main effect of timepoint *F*_(2,20)_ = 0.05, *P* = 0.31, interaction *F*_(2,20)_ = 1.2, *P* = 0.31). Data shown are mean ± SEM, representative EPSPs (scale bars = 2 mV/100 ms), raw EPSP amplitudes for individual experiments. *N* = 6 cells from 6 animals.

The D1-like receptor agonist SKF81297 (0.5 μM) produced a small, reversible increase in the amplitude of both inputs (Fig. 6*A*, see Supplementary Fig. S6*A*). Ten-micromolar SKF81297 also reversibly enhanced responses in both inputs but this was statistically insignificant (Fig. 6*B*, see Supplementary Fig. S5*A*). Ten-micromolar SKF81297 had no effect on paired-pulse ratio (100-ms inter-stimulus-interval [ISI]) in either pathway (see Supplementary Fig. S5*B*). Previous reports have argued that dopamine receptor expression is tightly linked to intrinsic cellular properties, with L5 pyramidal neurons that express D2-like receptors showing prominent I_h_ and D1-expressing neurons lacking prominent I_h_ (Gee et al. 2012). However, we found no relationship between the magnitude of I_h_ and modulation by D1R agonists (see Supplementary Fig. S6*C*), indicating that cellular heterogeneity does not account for the lack of effect of D1R activation.

We also found that activation of D2R-like receptors by quinpirole (10 μM) did not modulate ReRh or HPC inputs (Fig. 6*C*, see Supplementary Fig. S6*D*) and again no relationship was found between I_h_ and the effect of D2R activation (see Supplementary Fig. S6*E*).

### ReRh and HPC Inputs Undergo Associative Synaptic Plasticity via NMDA Receptor Activation

**Figure 7:**
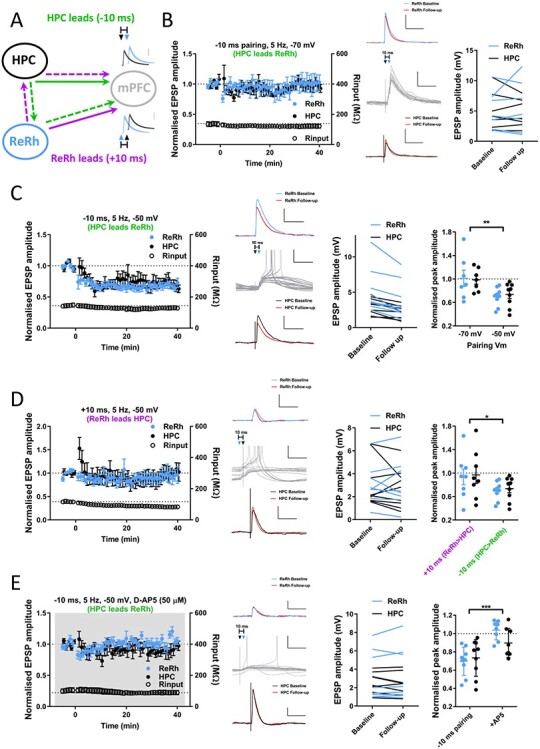
Pairing of HPC and ReRh inputs repeated at theta frequency induces NMDA receptor-dependent, associative LTD. (*A*) Schematic diagram showing hypothesized tripartite circuit dynamics. Information arising in HPC (green pathway) may project directly to mPFC (solid arrow) and feed forward disynaptically via ReRh (dashed arrows), HPC EPSPs would therefore precede ReRh in mPFC resulting in negative lag. Conversely, a signal originating in ReRh may reach mPFC directly and disynaptically via HPC (purple) resulting in the opposite temporal activation profile. (*B*) Theta-frequency pairing of HPC and ReRh inputs at −70 mV (HPC stimulus preceding ReRh by 10 ms, 100 pairs delivered at 5 Hz, Vm = −70 mV) does not induce synaptic plasticity in either pathway (Follow up: plasticity measured at 30–40 min after pairing; paired *t*-test: ReRh *t*_(6)_ = 0.6, *P* = 0.56; HPC *t*_(6)_ = 0.8, *P* = 0.45). Traces show example averaged EPSPs at baseline (ReRh; blue [above]/HPC; black [below]) and 30–40 mins (follow-up; red) and the first 15 pairings (gray; middle traces) with HPC stimulation denoted by black and ReRh by blue triangles, respectively. Scale bars EPSPs: 5 mV, 100 ms, pairing: 5 mV, 50 ms. Right: EPSP amplitudes for individual experiments. *N* = 7 cells from 7 animals. (*C*) Depolarization to −50 mV during 5 Hz pairing (−10 ms delay) induces LTD of ReRh and HPC inputs to PFC. Traces as in B except scale = 2 mV in non-pairing traces. Example experiment traces show baseline and 30–40 min EPSPs. Note incidence of spiking during pairing protocol. Individual experiment EPSP sizes at baseline and final 10 min (Wilcoxon signed ranks: ReRh Z = −2.7, *P* = 0.008; HPC Z = −2.7, *P* = 0.008). Normalized final EPSP amplitudes for −10 ms delay pairing performed at −70 and −50 mV (2-way ANOVA, effect of membrane potential: *F*_(1,28)_ = 11.3, *P* = 0.0023, main effect of pathway: *F*_(1,28)_ = 0.005, *P* = 0.95, interaction: *F*_(1,28)_ = 0.14, *P* = 0.7). *N* = 9 cells from 9 animals. (*D*) Pairing with +10 ms lag (ReRh precedes HPC by 10 ms) with depolarization does not induce plasticity in either pathway (Wilcoxon signed ranks ReRh: Z = −0.14, *P* = 0.89, HPC: Z = −0.42, *P* = 0.67). Traces as in (*B*). Normalized final EPSP amplitudes for +10 ms and − 10 ms at −50-mV membrane potential (2-way ANOVA, main effect of pairing order: *F*_(1,30)_ = 5.2, *P* = 0.030, main effect of pathway: *F*_(1,30)_ = 0.12, *P* = 0.73, interaction: *F*_(1,30)_ = 0.0002, *P* = 0.99). *N* = 8 cells from 7 animals. (*E*) Bath application of NMDA receptor antagonist D-AP5 (50 μM), as indicated by gray shading, blocks induction of LTD by −10 ms pairing. Traces as in (*B*), except EPSP scale bars = 1 mV. (Wilcoxon signed ranks ReRh: Z = −0.84, *P* = 0.4; HPC Z = −1.3, *P* = 0.21). Normalized amplitudes for −50 mV pairing in absence or presence of D-AP5 (2-way ANOVA, main effect of drug: *F*_(1,.30)_ = 21.0, *P* < 0.0001, main effect of pathway: *F*_(1,30)_ = 0.9, *P* = 0.35, interaction: *F*_(1,30)_ = 2.6, *P* = 0.12). *N* = 8 cells from 8 animals.

Since both ReRh and HPC inputs to mPFC are required for memory encoding we next asked whether ReRh inputs to mPFC undergo synaptic plasticity and whether ReRh and HPC inputs interact to induce synaptic plasticity. We hypothesized that a signal originating in either HPC or ReRh would project directly to mPFC and di-synaptically to mPFC via the other region, thus resulting in a short lag between inputs (Fig. 7*A*). We therefore paired optogenetic stimulation of ReRh and electrical stimulation of HPC afferents at time windows predicted from a simplified HPC-ReRh-mPFC circuit (Dolleman-van der Weel et al. 2019). HPC and ReRh stimuli were paired with 10-ms ISIs. Comparable patterns of activity have been shown to induce so called input-timing dependent plasticity (ITDP) in the HPC (Dudman et al. 2007). The pairing of HPC and ReRh inputs was performed at 5 Hz since HPC-mPFC coherence in the theta range has been associated with performance of working memory tasks (Jones and Wilson 2005; Siapas et al. 2005) and theta coherence can be enhanced following learning (Benchenane et al. 2010). ReRh plays a role in coordination of HPC-mPFC oscillations at theta (Hallock et al. 2016; Kafetzopoulos et al. 2018, though see: Roy et al. 2017) and delta (2–5 Hz; Roy et al. 2017) and contains cells with spontaneous firing across these ranges (Walsh et al. 2017).

We performed pairing of ReRh and HPC (10-ms ISI) repeated (100 pairs) at 5 Hz with the cell held at −70-mV membrane potential. Pairing consisted of either HPC preceding ReRh (−10 ms lag), or ReRh preceding HPC (+10 ms lag). Under these conditions pairing was exclusively subthreshold in all cells and did not result in plasticity when HPC led ReRh (−10 ms) or vice versa (+10 ms; Fig. 7*B*, see Supplementary Fig. S7*A*). The same results were obtained when ISI was reduced to ±5 ms (see Supplementary Fig. S7*A*). As ReRh has also been proposed to be involved in coordinating slow oscillations in the 0.1–1-Hz band (Dolleman-van der Weel et al. 2019) and ITDP delivered at 1 Hz induces plasticity in HPC (Dudman et al. 2007), we repeated ITDP protocols at 1 Hz, however these again failed to induce synaptic plasticity regardless of the pairing windows used (see Supplementary Fig. S7*A*).

To increase NMDA receptor activation, we modified the 5-Hz pairing protocol by depolarizing cells to −50 mV by intracellular current injection for the duration of the pairing. Under these conditions when HPC fibers were stimulated 10 ms before ReRh (−10-ms lag), pairing produced LTD in both ReRh (*P* = 0.008) and HPC (*P* = 0.008) inputs (Fig 7*C*) and was significantly different to the same protocol applied at −70 mV (2-way ANOVA of pairing at −70 and −50 mV: main effect of membrane potential *P* = 0.0023). In addition, when pairing was delivered at −50 mV a modest number of spikes was observed (see Supplementary Fig. S7*D*; 21.9 ± 7.5 spikes from 100 pairings, compared with none at −70 mV).

Next, we tested the hypothesis that plasticity depends on the temporal order of synaptic inputs by reversing the order of stimulation such that ReRh preceded HPC by 10 ms (+10 ms). Surprisingly, this protocol did not induce plasticity in either input (Fig. 7*D*; ReRh *P* = 0.89, HPC *P* = 0.67, 2-way ANOVA of +10 vs. −10 ms: main effect of pairing order: *P* = 0.030), this was not explained by a change in number of spikes fired (see Supplementary Fig. S7*D*). These data show that associative plasticity of ReRh and HPC synaptic inputs into mPFC critically depends on the temporal order of the incoming afferents.

Having shown that unidirectional pairing induces LTD in depolarized cells, each pathway was then stimulated alone at −50 mV. Stimulation of either HPC (see Supplementary Fig. S7*B*; *P* = 0.23) or ReRh fibers alone (see Supplementary Fig. S7*C*; *P* = 0.40) did not induce plasticity in the test or control pathway, confirming that this form of plasticity is associative in nature.

Associative plasticity of ReRh and HPC is dependent upon depolarization of the postsynaptic cell to −50 mV, resulting in spiking and presumably greater NMDA receptor activation. To identify the mechanisms by which plasticity is mediated we therefore paired HPC and ReRh stimulation (−10 ms) at −50 mV in the presence of NMDA receptor antagonist D-AP5 (50 μM; Fig. 7*E*). Neither ReRh (*P* = 0.4) nor HPC (*P* = 0.21; 2-way ANOVA of −10-ms pairing vs. D-AP5 data, main effect of drug: *P* < 0.0001) underwent plasticity in the absence of NMDAR activity. No significant decrease in spiking was observed due to the presence of D-AP5 (see Supplementary Fig. S7*D*). Together these results show that during depolarization, ReRh and HPC inputs interact in a unidirectional manner via NMDAR-mediated transmission to induce an associative form of synaptic plasticity at layer V mPFC pyramidal neurons.

## Discussion

Thalamic reuniens and rhomboid nuclei have emerged as additional important brain regions for performance of higher order-cognitive tasks which require HPC–mPFC interactions. Here we advance the understanding of this circuit, showing that ReRh and HPC inputs converge onto L5 pyramidal neurons in prelimbic cortex, these inputs undergo markedly different short-term plasticity and neuromodulation via muscarinic ACh receptors, and interact with specific timing and directionality to induce associative synaptic plasticity, revealing a potential memory encoding mechanism.

Anatomical evidence shows NRe axon labelling across all layers of mPFC (Vertes et al. 2006) and in vivo field recordings have recorded large amplitude EPSPs in both superficial and deep layers of prelimbic cortex following NRe stimulation (Viana Di Prisco and Vertes 2006; Eleore et al. 2011). Our data show that a high proportion (68%) of L5 pyramidal neurons receive input from ReRh and demonstrate with layer and cell-type specificity that ReRh fibers synapse directly onto L2/3 and L5 pyramidal cells. These data utilizing combinatorial activation of HPC and ReRh synapses onto the same pyramidal cells advance current understanding of the tripartite HPC–ReRh–mPFC circuitry. To achieve these advances, we used electrical stimulation of HPC inputs and optogenetic stimulation of ReRh inputs. Electrical stimulation between the dorsal tenia tecta and the nucleus accumbens in mPFC slices selectively activates HPC fibers as shown previously (Parent et al. 2010; Banks et al. 2015). To study HPC versus ReRh pathways using a within-cell design with all-optogenetic activation would require use of a second optogenetic construct with red-shifted activation wavelength (Klapoetke et al. 2014); however, such constructs have different channel properties to their blue-light activated counterparts and would therefore introduce further variables as well as potentially suffering from cross-pathway activation. Our viral injection of ChR2 in midline thalamus transduced neurons in reuniens and rhomboid nuclei and it was not possible based on current methodology to distinguish between inputs into mPFC from these 2 nuclei. Nevertheless, light stimulation resulted in EPSPs that were blocked by TTX and, under control stimulation intensities, were not rescued by 4-AP. This suggests that these ReRh EPSPs are action potential dependent with no, or minimal, direct opsin-dependent release. Furthermore, we show that there is no difference in short-term plasticity, effects of CCh, or release probability between electrical and optical stimulation of HPC afferents onto mPFC pyramidal cells. Therefore, the electrical/optical stimulation techniques used in the present study represent the most pragmatic way to directly compare HPC and ReRh synapses onto the same cell.

Previous studies have coalesced electrophysiological and anatomical data to show that L5 pyramidal neurons with different projection targets have different intrinsic membrane properties (Dembrow et al. 2010; Gee et al. 2012; Dembrow et al. 2015; Anastasiades et al. 2018). In the present study we observed small but statistically significant differences between the parameters of cells receiving HPC only inputs versus those receiving ReRh input alone or ReRh and HPC inputs (lower input resistance and larger Ih-mediated sag). In this respect the cells which receive only HPC input are electrophysiologically more akin to PT than IT cells. However, the differences we report are much smaller and have large variance, compared with those reported previously for IT and PT cells (Dembrow et al. 2010) and as the largest proportion of cells receiving HPC input are represented by the cells receiving both inputs, it seems unlikely that either ReRh or HPC inputs strongly targets layer 5 pyramidal neuron subtypes (though see: Dembrow et al. 2015; Liu and Carter 2018). Therefore, the precise targets of these inputs and how these impact local and extended circuitry warrants further examination.

### Short-Term Plasticity of ReRh-mPFC Transmission

A novel finding of the current study is that at theta-frequencies, which correspond to the instantaneous firing rate of NRe matrix cells (Walsh et al. 2017), ReRh input to both L2/3 and L5 pyramidal cells undergoes strong short-term depression (Fig. 3*A*, *B*). This contrasts the facilitation reported in in vivo studies (Viana Di Prisco and Vertes 2006; Eleore et al. 2011). However, in those studies intra-thalamic stimulation was delivered, which may mean that NRe neurons, rather than the synapses to mPFC, as in our study, are the locus of the reported facilitation. In vitro data from a study of matrix thalamus to mPFC L2/3 pyramidal neurons (Cruikshank et al. 2012) reported weak facilitation at 10 Hz; however only 1 of 11 animals in the data set reported had NRe ChR2 expression, with the remainder largely restricted to VM and AM thalamus, which have previously been reported to facilitate (Collins et al. 2018). Thus, methodological differences may explain the short-term plasticity findings across different studies. Our ReRh data more closely resemble short-term depression seen from the mediodorsal thalamus (Collins et al. 2018). HPC inputs to mPFC meanwhile do not undergo notable short-term plasticity at theta frequency whether by electrical or optical stimulation (Fig. 3*A*), in keeping with previous findings (Liu and Carter 2018).

What mechanism underlies short-term depression of ReRh inputs? We demonstrate that HPC ChETA_TC_ stimulation replicated electrical stimulation of HPC afferents at 5 and 10 Hz (Fig 3*C*). It is not possible to electrically stimulate ReRh-mPFC synapses selectively in vitro but the above control experiments for HPC-mPFC synapses show that it is extremely unlikely that artifacts of optogenetic stimulation or viral transduction of ReRh (Jackman et al. 2014) underlie the observed ReRh short-term depression. Differential AMPAR or NMDAR expression does not explain differences in summation as measures of transmission via these receptors did not differ. Furthermore, influence of mGlu, GABA_B_, nACh, or presynaptic NMDA receptors does not appear to underlie ReRh short-term dynamics as blocking these receptors had no effect on theta-frequency synaptic transmission. Use dependent blockade of NMDAR currents with MK801 suggest that the short-term depression seen in ReRh inputs is due to a high probability of release, as seen in other thalamocortical synapses (Gil et al. 1999). Furthermore, the similarity of the MK-801 block in electrical and optical stimulation of HPC afferents shows that the ReRh results are not due to an optogenetic artifact. In this respect ReRh projections are alike those of primary sensory thalamus and MD-mPFC projections (Sherman 2016; Collins et al. 2018).

The functional consequences of short depression of ReRh inputs to mPFC are not known but during spatial working memory, analysis of the directionality of HPC-mPFC theta coherence has been shown to change from HPC leading mPFC during the delay phase, to mPFC leading HPC during a decision phase (Hallock et al. 2016). As ReRh and mPFC are organized as a reciprocal loop, weakening of thalamocortical signal may allow for changes in the directionality of information transfer, enabling mPFC–ReRh–HPC communication to predominate. ReRh-mPFC depression may therefore facilitate the transitions between bottom-up and top-down processing, during which mPFC sends information back to HPC via NRe (Ito et al. 2015).

### Neuromodulation of ReRh and HPC Inputs

Cholinergic modulation of ReRh and HPC synapses onto mPFC cells was strikingly different. Previous data have shown cholinergic activation of mPFC with CCh produces depression of locally evoked excitatory transmission (Huang and Hsu 2010; Caruana et al. 2011; Martin et al. 2015) and of HPC synapses onto both L2/3 and L5 pyramidal cells (Wang and Yuan 2009; Ghoshal et al. 2017; Maksymetz et al. 2019). Here, we find that lower concentrations of CCh than used in the above studies acting via M2 muscarinic receptors produced acute depression of HPC input to L5 pyramidal cells.

In contrast to HPC transmission, ReRh inputs were unaffected by CCh. This is surprising since ReRh is abundant in both muscarinic and nicotinic receptors (Wamsley et al. 1984; Clarke et al. 1985) and infusion of muscarinic or nicotinic receptor antagonists into ReRh results in deficits of associative recognition memory encoding (Barker and Warburton 2018). Other corticothalamic synapses have been shown to be potentiated by addition of nAChR agonists (Gil et al. 1997) and nicotinic agonists have been shown to increase spontaneous excitatory transmission in mPFC, an effect which is absent from animals with extensive thalamic lesions (Lambe et al. 2003). It is possible that the relatively slow bath application of CCh used in the present study does not capture the effects of rapidly desensitizing nAChRs. However, muscarinic-LTD is also absent in MD projections to mPFC L5 (Maksymetz et al. 2019), therefore suggesting that insensitivity to muscarinic LTD may be a feature of thalamic inputs to mPFC. If ReRh-mPFC synapses are not depressed by ACh, then this ensures a mechanism that favors the ReRh inputs to mPFC over HPC inputs during periods of ACh signaling. The consequences of this shift in balance for mPFC circuitry and for synaptic plasticity at these 2 inputs remain to be determined, but will likely have implications for the mechanisms underlying encoding and retrieval of associative memory.

Previous reports have shown varied effects of dopaminergic ligands on glutamatergic transmission in mPFC, including both potentiation and depression of AMPAR and NMDAR mediated components, mediated via D1Rs and D2Rs (Law-Tho et al. 1994; Gao et al. 2001; Seamans et al. 2001; Urban et al. 2002; Gonzalez-Islas and Hablitz 2003; Banks et al. 2015). In this study we did not find strong evidence for modulation of either ReRh or HPC AMPA transmission by D1R or D2R agonists. In similar experiments we have previously shown that HPC-mPFC NMDARs undergo D2R-dependent depression (Banks et al. 2015) but there was no direct effect on AMPAR transmission (though see: Burke Jr et al. 2018). Therefore rather than modulating long-range synaptic transmission, dopamine’s role in the mPFC may lie in other mechanisms not explicitly tested in this study, such as its effects on local synaptic transmission (Burke Jr et al. 2018), cell excitability (Gee et al. 2012; Anastasiades et al. 2019), and modulation of synaptic plasticity (Otani et al. 2003).

### ITDP of ReRh and HPC Synapses

Here we report a novel form of associative plasticity at both the HPC and ReRh synapses induced by pairing of these inputs, but only when HPC leads ReRh. The plasticity protocol was designed to be physiologically plausible, with the timing windows predicted on a simplified version of the HPC-ReRh-mPFC tripartite circuit (Dolleman-van der Weel et al. 2019). Synaptic plasticity was not induced at RMP but was induced at −50 mV and was blocked by D-AP5, suggesting that ReRh and HPC synapses interact via NMDARs to induce plasticity. In this study therefore NMDAR activation resulting from depolarization is essential for associative plasticity. What mechanisms may be responsible for producing depolarization in vivo? It is known that at higher frequencies HPC EPSPs show marked facilitation (Liu and Carter 2018) and therefore could provide the depolarization required. Gamma frequency oscillations during associative memory might therefore be important in coordinating ReRh inputs with depolarization mediated by HPC inputs. Whilst experiments in this study were conducted using theta frequency stimulation, in future it would be interesting to determine if, at resting potential, pairing HPC with ReRh at higher frequencies induces similar synaptic plasticity. In addition, it is possible that activation of neuromodulatory systems may result in direct depolarization of mPFC neurons (Haj-Dahmane and Andrade 1996; Tikhonova et al. 2018), thus contributing to induction of pairing induced NMDAR-dependent plasticity at HPC and ReRh synapses in mPFC.

In cortex, NMDA receptors are tetramers composed of 2 obligatory GluN1 subunits and any permutation of 2 GluN2A/GluN2B subunits. GluN2B containing receptors, which have slower kinetics than those only expressing GluN2A subunits (Paoletti et al. 2013), are expressed at higher levels in adult mPFC compared with other cortical regions (Wang et al. 2008) including at HPC afferents (Flores-Barrera et al. 2014). Our findings show that ReRh and HPC synapses had equivalent levels of NMDAR expression and similar sensitivity to a GluN2B-selective antagonist, suggesting that both these inputs are abundant in GluN2B subunits, which is not a universal feature of synapses onto L5 pyramidal neurons (Flores-Barrera et al. 2014). GluN2B subunit kinetics facilitate sustained charge-transfer, and therefore Ca^2+^ influx, at low frequency (Erreger et al. 2005) which contributes to GluN2B-dependent LTD (Massey et al. 2004). Slow NMDAR activity may be conducive to interaction of the spines of ReRh and HPC synapses via spatiotemporal summation with possible mechanisms for plasticity induction including activation of calcium-dependent second messengers, release of calcium from intracellular stores (Dudman et al. 2007) or generation of dendritic calcium spikes (Larkum 2013). Although spiking was elicited by −10-ms pairing at −50 mV the number of spikes did not correlate with the induction of plasticity suggesting that action potentials are not a critical factor in induction of ITDP, as has also been described for ITDP in other brain regions (Dudman et al. 2007; Williams and Holtmaat 2019).

ITDP of ReRh and HPC inputs is, to the best of our knowledge, distinctive in that plasticity was induced in both pathways, in contrast to that in other brain regions where only the more proximal of the 2 synaptic inputs undergoes plasticity (Dudman et al. 2007; Williams and Holtmaat 2019). This suggests that interaction of HPC and ReRh synapses during pairing occurs in overlapping dendritic components, thus promoting spread of NMDAR-mediated depolarization and equalizing Ca^2+^ influx between spines. Functional and anatomical data supports overlapping distribution of synaptic input from HPC (Liu and Carter 2018) and ReRh (Fig. 1*B*) in deep layers of mPFC. In addition, ReRh and HPC EPSPs show equal rise time (ReRh 3.6 ± 0.3 ms, HPC 3.7 ± 0.3 ms) indicating equal distance from the soma (Sjostrom and Hausser 2006).

However, the mechanism by which pairing induced plasticity when HPC stimulation preceded ReRh but not when pairing order was reversed are not clear. Possible explanation may involve differential activation of feed-forward inhibitory circuits, for example ReRh may recruit stronger feedforward inhibition than HPC which may impair summation between pathways when ReRh activation precedes HPC. Alternatively feedforward inhibition recruited by ReRh and HPC could target different subcellular locations to achieve the same effect (Cruikshank et al. 2012) or activation of HPC inputs could result in tightly timed disinhibition to allow plasticity with one direction of pairing but not the other (Williams and Holtmaat 2019). To fully understand which of these (combinations of) possibilities is important will require considerable further investigation.

Each of mPFC, HPC, and ReRh are required for many high order mnemonic and executive functions (Dolleman-van der Weel et al. 2019) including spatial navigation (Ito et al. 2015; Jankowski et al. 2015), associative recognition memory (Barker and Warburton 2018), and sequence memory (Jayachandran et al. 2019), but the circuit mechanisms underlying these functions are not understood. Whilst the behavioral function of the associative plasticity we describe remains to be determined, the specific timing conditions for plasticity we report suggest that ReRh may impose a timing control which determines the salience of HPC signals and promotes their encoding. ReRh has been noted to receive input from many other higher order regions including but not limited to entorhinal cortex, perirhinal cortex, and amygdala (Dolleman-van der Weel et al. 2019), and could act to integrate, for example, novelty or contextual information with incoming HPC spatial information which may promote encoding or consolidation of object-place associations (Barker and Warburton 2018). Such a mechanism may depend on ReRh to coordinate oscillatory activity across multiple regions, and the observation of a high incidence of connectivity between ReRh and L5 principal neurons in the present study may act to amplify signals from perirhinal and entorhinal cortices which have more sparse direct connections with prelimbic cortex (Hoover and Vertes 2007) but which are crucial for many forms of memory processing.

## Supplementary Material

SupplementaryFig1ccc_tgab029

SupplementaryFig2ccc_tgab029

SupplementaryFig3ccc_tgab029

SupplementaryFig4ccc_tgab029

SupplementaryFig5ccc_tgab029

SupplementaryFig6ccc_tgab029

SupplementaryFig7ccc_tgab029

Supplementary_material_Banks_et_al_CCC_tgab029

## Notes

We would like to thank Jack Mellor, Paul Anastasiades, and members of the Bashir and Warburton laboratories for contributions, helpful discussion and comments on the manuscript, and Dr Clair Booth for assistance with MATLAB analyses and mCherry staining. *Conflict of Interest*: None declared.
