# Supplementary figures and images for: Plasticity in Prefrontal Cortex Induced by Coordinated Synaptic Transmission Arising from Reuniens/Rhomboid Nuclei and Hippocampus
Source: Cereb Cortex Commun. 2021 Apr 14;2(2):tgab029. doi: 10.1093/texcom/tgab029 (PMC8152950; doi:10.1093/texcom/tgab029)

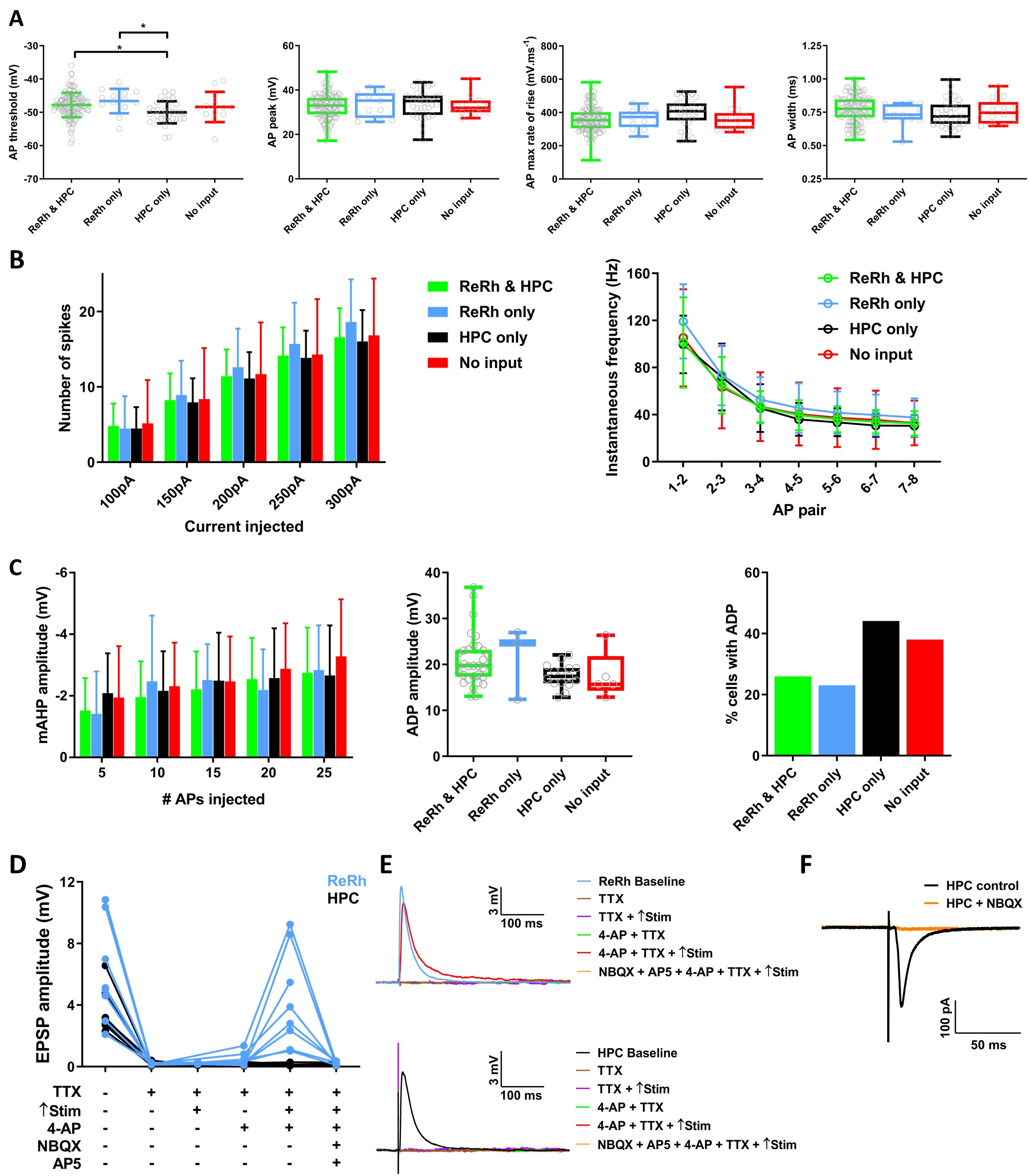

Supplement: SupplementaryFig1ccc_tgab029 [file supplementaryfig1ccc_tgab029.jpeg]

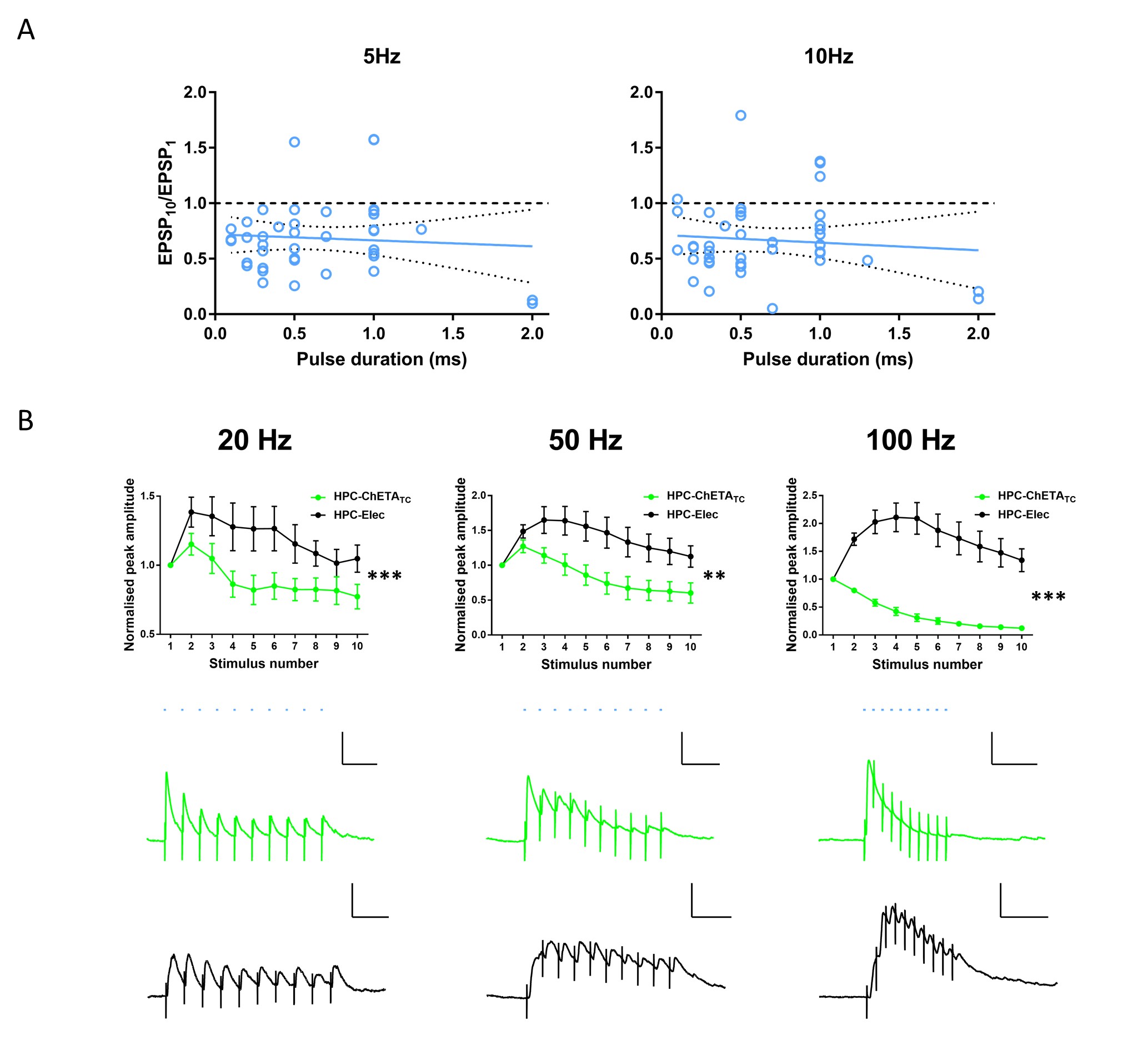

Supplement: SupplementaryFig2ccc_tgab029 [file supplementaryfig2ccc_tgab029.jpeg]

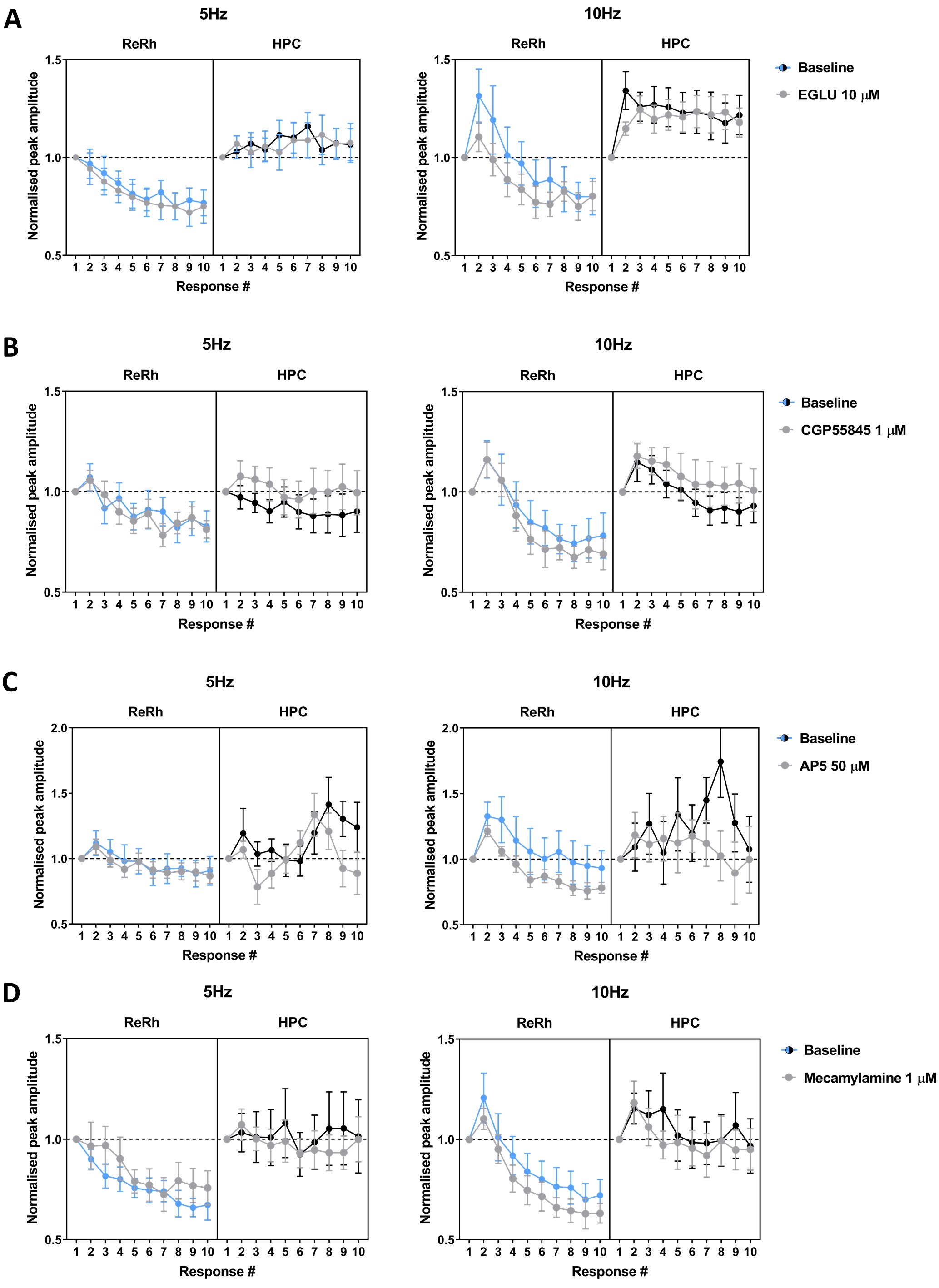

Supplement: SupplementaryFig3ccc_tgab029 [file supplementaryfig3ccc_tgab029.jpeg]

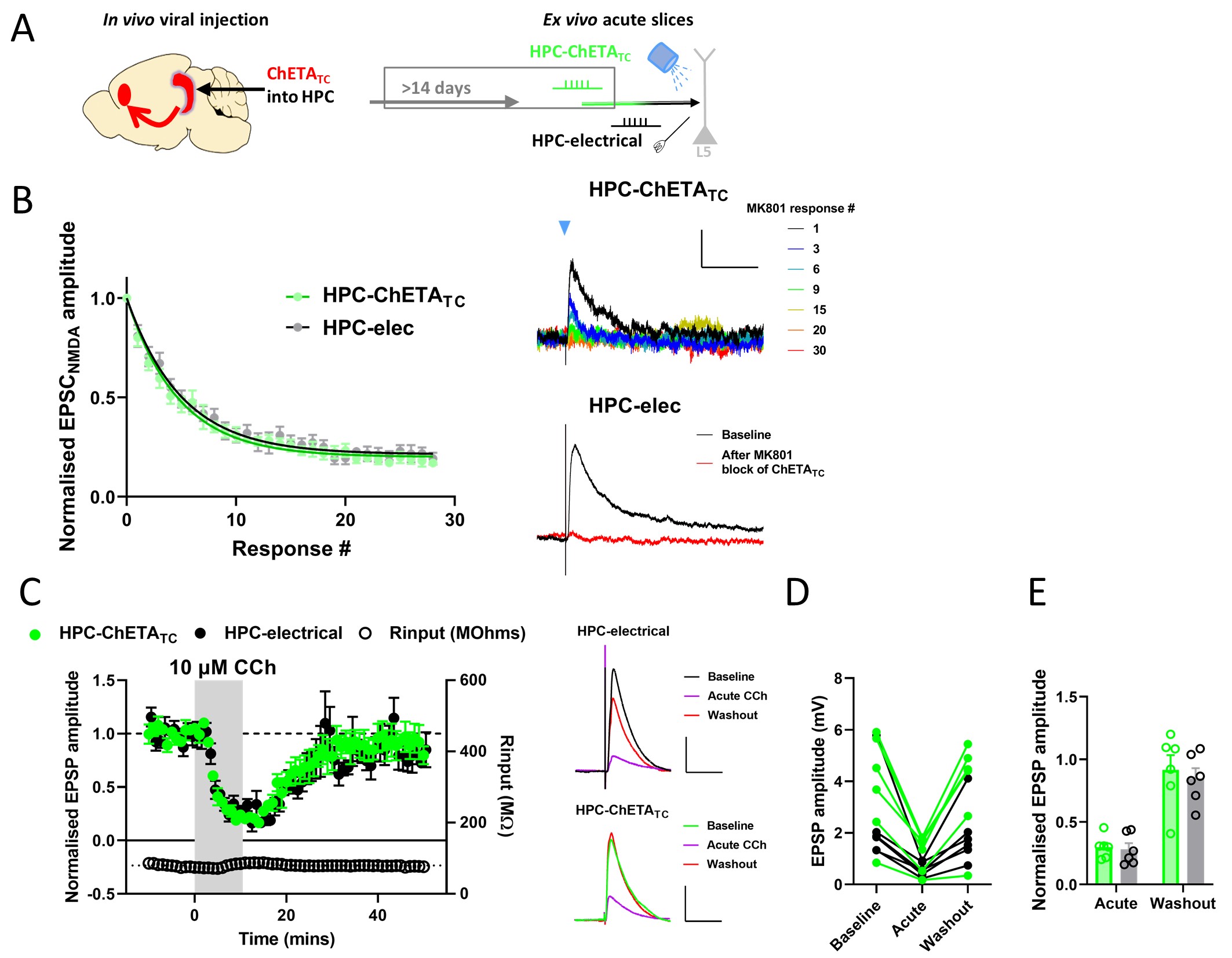

Supplement: SupplementaryFig4ccc_tgab029 [file supplementaryfig4ccc_tgab029.jpeg]

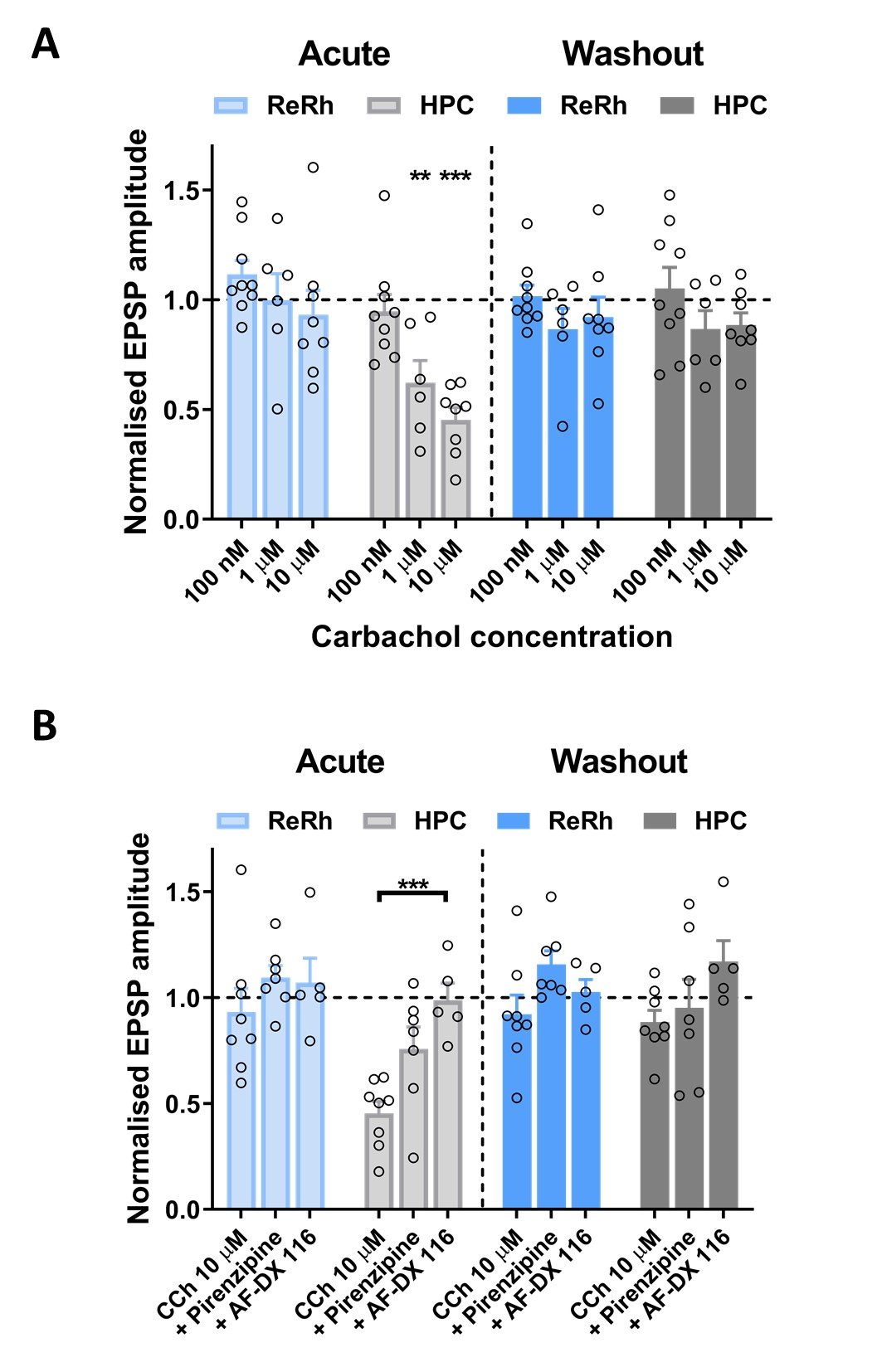

Supplement: SupplementaryFig5ccc_tgab029 [file supplementaryfig5ccc_tgab029.jpeg]

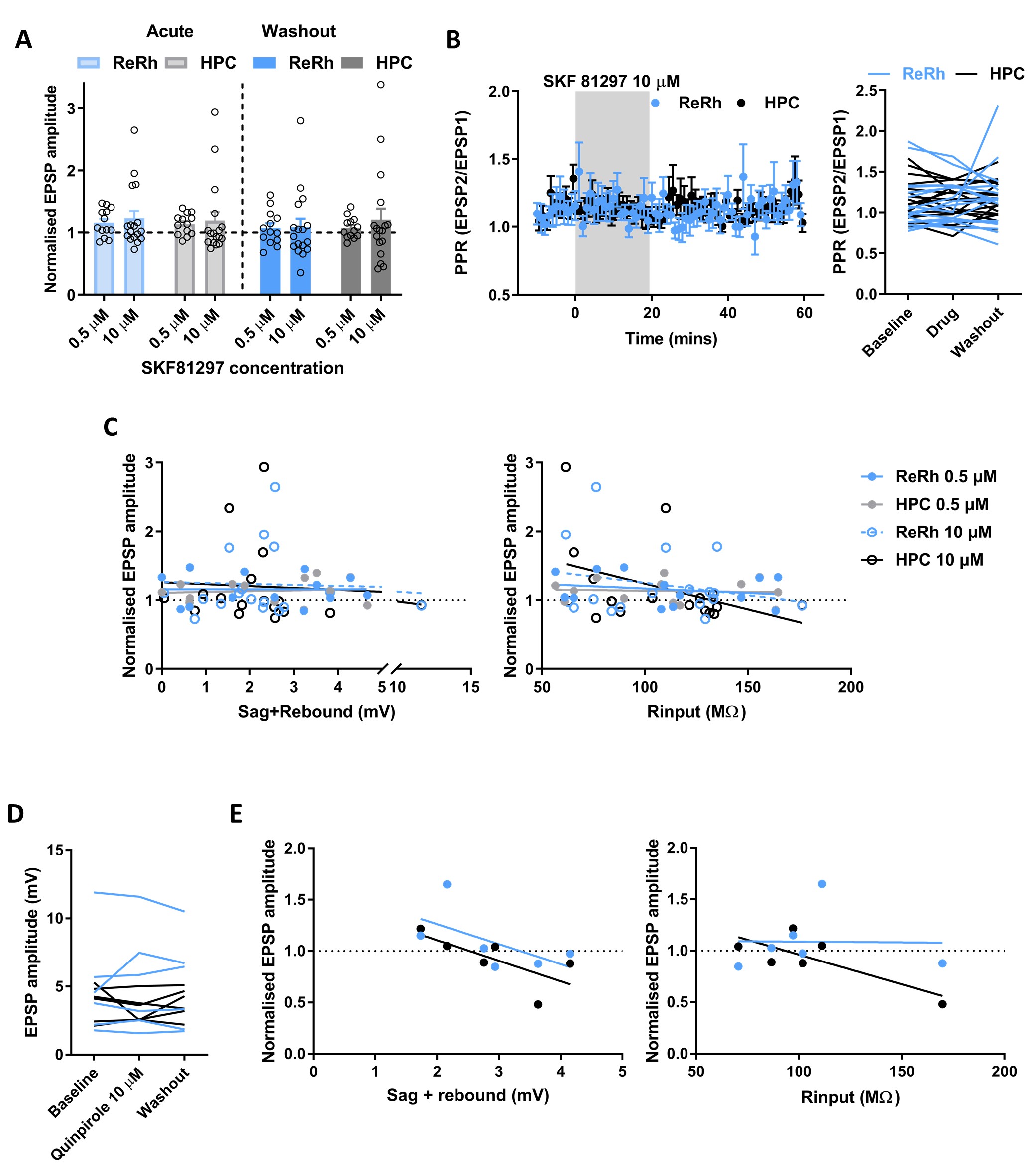

Supplement: SupplementaryFig6ccc_tgab029 [file supplementaryfig6ccc_tgab029.jpeg]

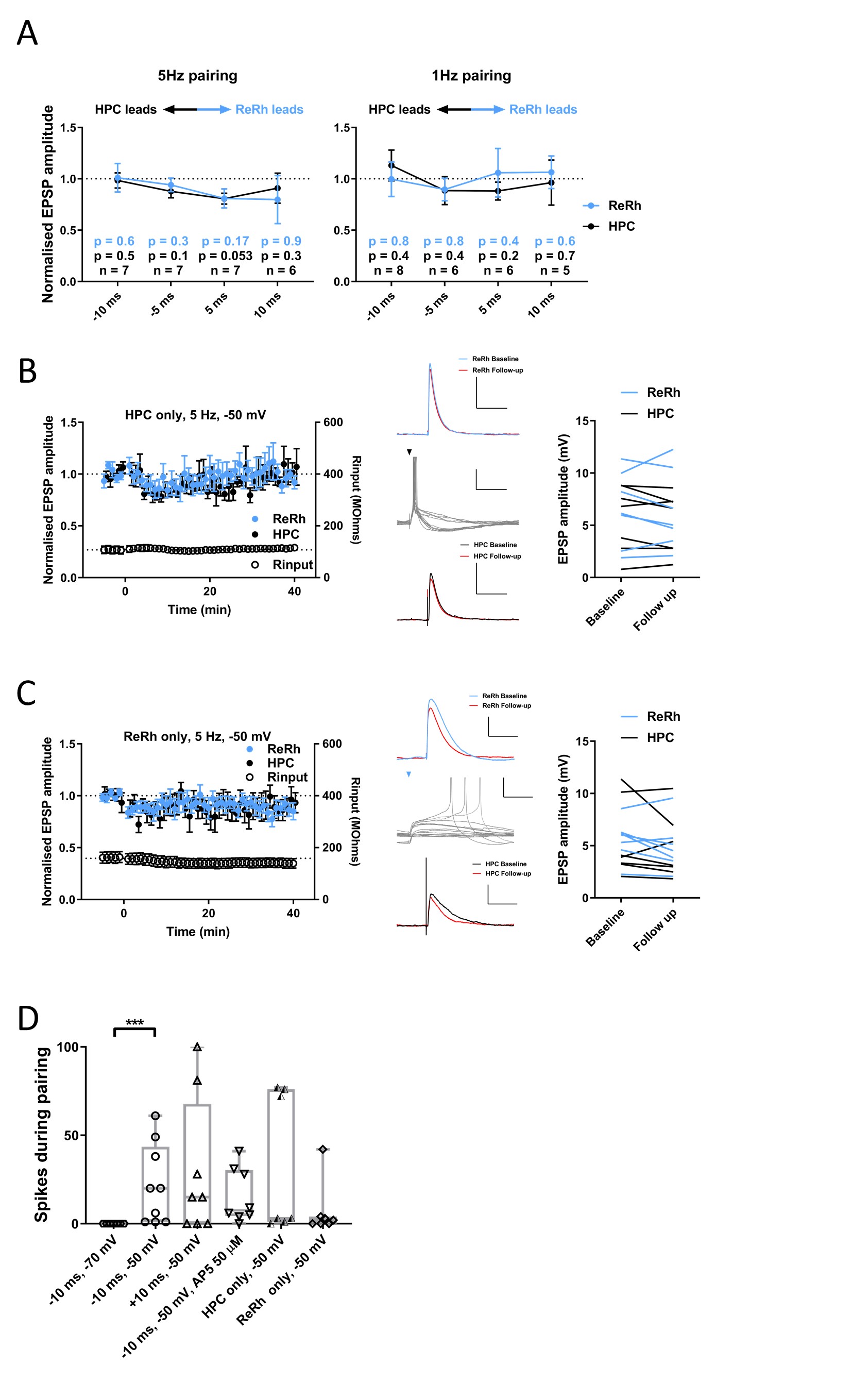

Supplement: SupplementaryFig7ccc_tgab029 [file supplementaryfig7ccc_tgab029.jpeg]
