# Supplementary material for: Plasticity in Prefrontal Cortex Induced by Coordinated Synaptic Transmission Arising from Reuniens/Rhomboid Nuclei and Hippocampus
Source: Cereb Cortex Commun. 2021 Apr 14;2(2):tgab029. doi: 10.1093/texcom/tgab029 (PMC8152950; doi:10.1093/texcom/tgab029)
Supplement: Supplementary_material_Banks_et_al_CCC_tgab029 [file supplementary_material_banks_et_al_ccc_tgab029.docx]

**Supplementary Figure 1**

**A** – Action potential properties measured from first spike fired at the lowest current injection level which produced spiking in Fig S1B. Threshold: ANOVA F_(3,183)_= 4.0, p = 0.0085, * p < 0.05 Tukey’s multiple comparisons. Other parameters tested using Kruskal-Wallis due to one or more column failing Shapiro-Wilk test for normality. Peak, p = 0.66. Max rate of rise, p = 0.069. Width, p = 0.19.

**B –** Left panel, number of spikes fired in response to 500 ms depolarising current injections of varying amplitudes, 2-way ANOVA main effect of synaptic input F_(3,183)_ = 0.43, p = 0.74. Right, instantaneous firing frequency for first 7 action potential pairs, repeated-measures ANOVA between subjects effect of synaptic input F_(3,182)_ = 1.2, p = 0.30. Data shown as mean ± SD, n as indicated in Fig 1E.

**C** – Left panel, medium afterhyperpolarisation (mAHP) amplitude following fixed numbers of action potentials. Between subject effects: F_(3,182)_ = 1.0, p = 0.38. Centre: afterdepolarisation (ADP) amplitude following single action potentials evoked by 2000 pA, 2 ms current. Kruskal-Wallis p = 0.085. Right, percentage of cells in each group with prevalent ADP, Pearson χ^2^ = 4.95, p = 0.18.

**D** – Basal ReRh and HPC EPSPs are blocked by bath application of tetrodotoxin (0.5 µM). Increasing stimulus strength or application of 4-AP (100 µM) alone were insufficient to rescue ReRh transmission, however increased stimulation strength in the presence of 4-AP restored ReRh transmission. These data are consistent with basal optogenetic stimulation parameters producing action-potential dependent EPSPs, with increased stimulus strength required to achieve over-bouton release in 4-AP. ReRh EPSPs were completely blocked by glutamate antagonists. Note that electrically evoked HPC EPSCs are not inducible even in the presence of 4-AP. ReRh stimulus strength was achieved by increasing light pulse duration to 5ms from baseline values of (0.2-2ms). HPC stimuli were doubled in amplitude compared to baseline.

**E** – EPSP traces from an example experiment in (**D**).

**F** – HPC EPSC blocked by bath application of 5 µM NBQX.

**Supplementary Figure 2 – Optogenetic stimulation for high-frequency synaptic transmission**

**A** Short-term depression of ReRh EPSPs at 5 and 10 Hz shows no relationship to duration of light pulses used to excite ChETA_TC_-expressing ReRh afferents. Blue line shows linear regression, dotted lines 95% confidence intervals (slope: 5 Hz = -0.054 ± 0.11, 10 Hz = -0.069 ± 0.12). Data taken from baselines shown in Fig 3A, 4B, and S3A-D, n = 42.

**B** Comparison of HPC afferent electrical and optogenetic stimulation. Top row: mean ± SEM of normalised amplitudes showing that ChETA_TC_ was unable to reproduce the short-term plasticity of electrical stimulation at 20 Hz (main effect of stimulation method 20 Hz: F_(1,12)_ = 10.3, p = 0.008; response number: F_(2.2,26.9)_ = 14.8, p = 3 x 10^-5^; interaction: F_(2.7,32.2)_ = 3.2, p = 0.041), 50Hz (stimulation method F_(1,12)_ = 9.45, p = 0.01; response number F_(1.6,19.2)_ = 21.2, p = 3 x 10^-5^; interaction F_(2.1,24.7)_ = 4.8, p = 0.016) or 100 Hz stimulation (stimulation method F_(1,12)_ = 30.2, p = 0.0003; response number F_(1.4,14.1)_ = 12.1, p = 0.002; interaction F_(1.7,17.3)_ = 6.3, p = 0.011; n = 13). Bottom row: examples traces of optogenetic (green) and electrical (black traces) from same cell. Note lack of subsequent peaks after the first optogenetic response at 100 Hz, accentuated by the pronounced LED-artefacts in this recording. Blue squares indicate LED activation. Scale bars = 3 mV/50 ms except 20 Hz, where x-axis is 100 ms.

**Supplementary Figure 3 – Antagonism of group II mGlu, GABA_B_, NMDA or nicotinic receptors does not affect short-term plasticity of ReRh or HPC synapses at theta frequencies**

**A** Bath application of group II mGluR receptor antagonist EGLU (10 µM) did not affect transmission in either pathway at 5 or 10 Hz (5 Hz: ReRh F_(8,72)_ = 0.48, p = 0.87; HPC F_(3.1, 28)_ = 0.98, p = 0.42; 10 Hz: ReRh F_(3.3,29.9)_ = 2.8, p = 0.053, HPC F_(3.3, 29)_ = 2.55, p = 0.071). N = 10 cells from 10 animals.

**B** GABA_B_ receptor antagonist CGP55845 (1 µM) did not affect short-term plasticity of ReRh or HPC at 5 or 10Hz (5 Hz: ReRh F_(8,72)_ = 1.3, p = 0.28; HPC F_(1.9,17.3)_ = 0.51, p = 0.60; 10 Hz: ReRh F_(8,72)_ = 0.5, p = 0.86, HPC F_(2.9, 26.2)_ = 0.80, p = 0.50). N = 10 cells from 10 animals.

**C** NMDAR antagonist D-AP5 (50 µM) did not alter short-term plasticity at 5 or 10 Hz in either pathway (5 Hz: ReRh F_(1.9,11.2)_ = 1.1, p = 0.38; HPC F_(1.9,9.6)_ = 0.45, p = 0.64; 10 Hz: ReRh F_(1.5,8.9)_ = 2.5, p = 0.15, HPC F_(2.0, 10.1)_ = 1.8, p = 0.22). N = 7 cells from 6 animals.

**D** Bath application of nicotinic acetylcholine receptor antagonist mecamylamine (1 µM) did not affect transmission in either pathway at 5 or 10 Hz (5 Hz: ReRh F_(2.5,20.3)_ = 1.42, p = 0.27; HPC F_(7.6, 60.8)_ = 0.82, p = 0.52; 10 Hz: ReRh F_(3.1,24.4)_ = 0.35, p = 0.79, HPC F_(2.3, 18.7)_ = 0.9, p = 0.44). N = 8 cells from 8 animals.

For all statistical tests Greenhouse-Geisser corrections were applied when Maulchly’s test of sphericity was below 0.05.

**Supplementary Figure 4 – Electrical stimulation of HPC fibres has pathway specificity and optogenetic activation of HPC fibres does not affect their release probability.**

A – Animals were injected with AAV9-CaMKii-hChR2(E123T/T159C)-mCherry into the hippocampus and 14 or more days later acute mPFC slices were made. HPC🡪mPFC afferent inputs onto L5 pyramidal neurons was evoked by optogenetic and conventional electrical stimulation.

B – Isolated HPC-mPFC NMDA receptor mediated EPSCs evoked by either optogenetic (green) or electrical (greyscale) underwent activity-dependent block by MK-801 (40 μM) which occurred at the same rate (single exponential curve constrained to Y0, electrical τ = 5.01, ChETA_TC_ τ = 4.72, extra sum of squares F-test F(2,605) = 2.2, p = 0.11), demonstrating that optogenetic activation of HPC afferents does not alter their release probability. N_electrical_ = 10 cells from 9 animals, N_ChETA_ = 11 cells from 9 animals. Upper traces show example MK-801 blockade of optogenetically-evoked HPC-mPFC EPSC_NMDA_, coloured by stimulus number in presence of MK-801. Lower traces show, from the same cell, electrically evoked HPC NMDA_EPSC_ before MK-801 application and after the blockade of optogenetically evoked EPSC_NMDA_. The absence of pathway independence demonstrates that electrical stimulation specifically activates HPC fibres. Scale bars = 100 pA/100 ms.

C – Pooled data showing both electrically- and optogenetically-evoked HPC EPSPs undergo the same degree of short-term modulation by bath application of 10 µM CCh (n = 6 cells from 6 animals). Example traces show responses from the same cell at baseline (-10 to -1 mins), acute (10-19 mins) and washout (40-49 mins), scale bars = 2 mV/50 ms. Scatter of individual cells raw (D) and normalised (E) amplitudes. Two-way ANOVA main effect of stimulation method: F_(1,10)_ = 0.21, p = 0.66; timepoint: F_(1,10)_ = 101.0, p < 0.0001; interaction: F_(1,10)_ = 0.19, p = 0.68.

**Supplementary Figure 5 – Summary of cholinergic modulation of inputs to PFC**

**A**– Summary of CCh effects on normalised ReRh and HPC EPSPs at acute (10-19 mins) and washout (40-49 mins) timepoints. Bars = mean +SEM, circles = individual cells. Two-way repeated-measures ANOVA revealed a timepoint*pathway*concentration interaction, demonstrating that CCh selectively attenuates HPC, not ReRh inputs, in a reversible, concentration-dependent manner (main effects: pathway F_(1,20)_ = 0.5, p = 0.48; time-point F_(1.8,36)_ = 11.3, p = 0.001; concentration F_(2,20)_ = 1.0, p = 0.37; interactions: pathway x concentration F_(2,20)_ = 1.2, p = 0.31; timepoint x concentration F_(3.6,36)_ = 3.9, p = 0.011; pathway x timepoint F_(1.8,35)_ = 14.7, p = 0.00004; timepoint x pathway x concentration F_(4,40)_=3.0, p = 0.029). Post-hoc analysis with Sidak’s multiple comparison: **p = 0.0016; ***p = 0.0002. All other comparisons p > 0.05.

**B** – Summary of data shown in Fig4B & C. Two-way repeated-measures ANOVA (ReRh: main effect of drug F_(2,34)_ = 2.9, p = 0.07; timepoint: F_(1,34)_ = 0.0009, p = 0.98; interaction F_(2,34)_ = 0.18, p = 0.84; HPC: main effect of drug F_(2,34)_ = 9.5, p = 0.0005; timepoint: F_(1,34)_ = 12.8, p = 0.0011; interaction F_(2,34)_ = 1.3, p = 0.29. Sidak’s post-hoc analysis, *** p = 0.001. All other comparisons p > 0.05.

**Supplementary Figure 6 – Dopaminergic modulation of inputs to PFC**

**A -** Summary of SKF81297 data showing individual experiments normalised to baseline overlaid over mean ± SEM bar graph. Acute/washout are averaged amplitudes from final 10 mins of drug application and final 10 mins of recording.

**B** – Paired-pulse ratio (100 ms inter-stimulus-interval) is not altered by bath application of SKF81297 (10 µM). Two-way repeated-measures ANOVA: main effect of timepoint: F_(2,64)_ = 0.4, p = 0.7; pathway F_1,32)_ = 0.01, p = 0.9; interaction F_(2,64)_ = 0.2, p = 0.8. Left shows mean + SEM values, right shows individual experiment PPR in each pathway at baseline, final 10 minutes of drug application and final ten minutes of recording.

**C** –Acute effect of SKF81297 on synaptic strength plotted versus sum of sag and rebound in response to a -100 pA hyperpolarising current injection (left) or cell input resistance at start of recording (right). Slopes are not significantly different from 0 in any instance.

**D** – EPSP amplitudes in response to quinpirole for individual cells as shown in Fig 5C. ReRh and HPC pathways shown in blue/black, respectively.

**E** – Quinpirole (10 µM) data as for **D**. Slopes are not significantly different from 0 in any instance. ReRh and HPC pathways shown in blue/black, respectively.

**Supplementary Figure 7 – Input-timing dependent plasticity of ReRh and HPC does not occur at -70 mV and requires activation of both pathways**

**A** – Summary of pairing experiments showing mean + SEM normalised amplitude of ReRh and HPC EPSPs 30-40 mins after pairing with different lags as indicated. Left graph shows pairing delivered at 5 Hz, right at 1 Hz. All experiments were performed at -70 mV. Plasticity was absent in both pathways at all frequencies and pairing delays tested (paired t-test of raw EPSP amplitudes, p values displayed on graph in blue for ReRh and black for HPC). For all experiments n = number of cells, 1 cell per animal.

**B** – 5 Hz stimulation of HPC afferents at -50 mV (single stimuli) does not induce plasticity of test pathway (paired t-test, HPC t_(6)_ = 1.4, p = 0.23) or control pathway (ReRh, t_(6)_ = 0.4, p = 0.74). Traces show example averaged EPSPs at baseline (blue/black) and 30-40 mins (red) and 15 pairings (grey), stimulation denoted by triangle. Scale bars EPSPs: 5 mV, 100 ms, pairing: 10 mV, 50 ms. Right panel: raw EPSP amplitudes at baseline and final 10 mins. N = 7 cells from 7 animals.

**C** – 5 Hz stimulation of ReRh afferents at -50 mV (single stimuli) does not induce plasticity of test pathway (Wilcoxon signed ranks, ReRh Z = -0.84, p = 0.40) or control pathway (HPC, Z = 0.98, p = 0.33). Traces show example averaged EPSPs at baseline (blue/black) and 30-40 mins (red) and 15 pairings (grey), stimulation denoted by triangle. Scale bars EPSPs: 2 mV, 100 ms, pairing: 10 mV, 50 ms. Right panel: raw EPSP amplitudes at baseline and final 10 mins. N = 7 cells from 7 animals.

**D** – Total number of spikes fired for experiments shown in Fig 7 and Fig S6. Data point represent individual cells, box plots show median, inter-quartile range and whiskers show minima and maxima. Data were subject to Kruskal-Wallis test (KW statistic = 14.6, p = 0.012) and post-hoc Dunn’s multiple comparisons were carried out against vs -10 ms pairing at -50 mV, *** = 0.007, all other comparisons p > 0.05.
